# Bio-priming of tomato seedlings with bacterial consortium against *Fusarium oxysporum*: a study on morphological parameters and molecular profiling

**DOI:** 10.3389/fmicb.2025.1606896

**Published:** 2025-07-09

**Authors:** Keerthana Rangasamy, Arabi Mohammed Saleh

**Affiliations:** ^1^School of Bioscience and Technology, Vellore Institute of Technology, Vellore, India; ^2^VIT School of Agricultural Innovations and Advanced Learning, Vellore Institute of Technology, Vellore, India

**Keywords:** bacterial consortia, biological control, antioxidants, gene expression, metagenomics, antifungal activity

## Abstract

Soil-borne diseases significantly threaten global crop production, resulting in substantial economic losses. Among these, *Fusarium oxysporum*, a major pathogen responsible for wilt in the root zones, severely affects tomato (*Solanum lycopersicum*), a widely consumed yet vulnerable vegetable. Conventional management strategies rely on fungicides and synthetic chemicals, which pose environmental and health risks, prompting the exploration of safer alternatives such as plant growth-promoting rhizobacteria (PGRP). In this study, we investigated the efficacy of two bacterial isolates, *Pseudomonas aeruginosa* VITK-1 and *Burkholderia cepacia* VITK-3, both individually and as a consortium, in the presence of *Fusarium oxysporum* under greenhouse conditions. *In vitro* assays revealed that the isolates inhibited *Fusarium oxysporum*, with rates ranging from 64.1 to 76.5%. Additionally, significant inhibition was observed against *Ralstonia solanacearum*, *Septoria protearum* (57.2%), *Verticillium dahlia* (88.5 to 81%), and *Cercospora canescens* (66.1 to 47.7%) *in vitro*. Both strains produced bioactive compounds against the test pathogens and formed biofilms, which enhanced plant growth and suppressed phytopathogens. Consortium treatment with *Fusarium oxysporum* significantly improved tomato seedlings’ antioxidant activity, including superoxide dismutase (SOD), catalase (CAT), phenolic, and flavonoid content, along with enhanced physiological parameters. Gene expression analysis confirmed the up-regulation of defense-related genes, while metagenomic profiling indicated improvements in the soil microbial community under consortium treatment with *Fusarium oxysporum* compared to individual treatments and untreated controls. These findings underscore the potential of bacterial consortia as effective biocontrol agents that promote plant health and soil microbiome integrity.

## Graphical abstract

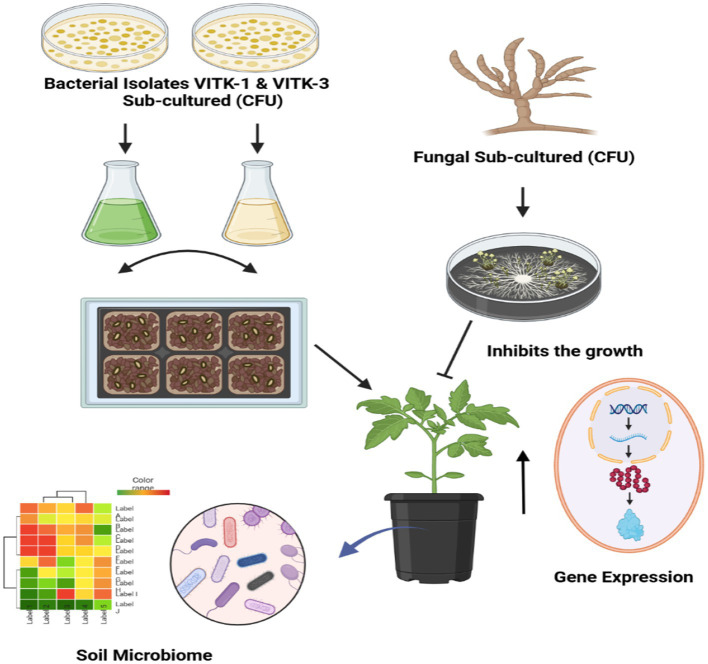

## 1 Introduction

*Solanum lycopersicum* (Tomato) is one of the most nutritious vegetables, available globally. It is a key source of carotenoids and has characteristics similar to other commonly cultivated crops like potatoes and yellow corn (Konuşkan, 2015). The tomato fruit pulp has natural antioxidant activity and is rich in vitamins A, and C. It is also a commonly cultivated crop, with production increasing substantially in countries like the United States and India with around 30% in 2017 alone, surpassing production targets (Mazzei et al., 2021). According to the FAOSTAT report (2024), India and China are the world’s leading tomato producers, contributing to a combined production of approximately 186 million tons over the 5 million hectares in 2022 (Flores and Poveda, 2025). However, tomatoes are vulnerable to soil-borne pathogens, which can negatively impact their growth and yield. Due to varying climatic conditions and humidity levels, tomatoes are vulnerable to across 200 soil-borne pathogens worldwide (Ma et al., 2023; Panno et al., 2021). These pathogens include fungi, bacteria, and viruses, which cause significant crop losses were estimated at 10–80%, and reduce the yield and nutritional value of tomatoes (Attia et al., 2020; Ma et al., 2023; Singh et al., 2017).

Soilborne pathogens such as *Fusarium oxysporum*, *Alternaria solani*, and *Ralstonia solanacearum* are particularly problematic, as they infect the plant rhizosphere, disrupting the relationship between the soil and the plant (Kashyap et al., 2021; Raaijmakers et al., 2009). The economic impact of these pathogens is substantial, reducing both the quality and quantity of tomato production globally (Attia et al., 2020; Mazzei et al., 2021). Notably, pathogens like *F. oxysporum* and *R. solanacearum* tend to induce diseases early in the plant’s growth, particularly at the seedling stage, by damaging the xylem of the host tissue (Di et al., 2016; Mozumder et al., 2022; Wang et al., 2023). Fungal and bacterial infections often trigger immune responses in the plant, causing cell death and, ultimately, a reduction in crop yield (Cao et al., 2018; de Lamo and Takken, 2020).

To combat these challenges, various solutions have been explored, including the use of chemical fertilizers and pesticides, which are typically applied before or after the seedling stage to protect plants from pathogens (de Lamo and Takken, 2020). However, because these chemicals pollute the environment, build up in the food chain, and pose health risks to humans, their continued use has sparked concerns (Agamy et al., 2013; Cao et al., 2018; López-Aranda et al., 2016). To address these challenges, plant growth-regulating microorganisms have been identified as prospective bio-control agents for treating pathogens like *F. oxysporum* and *R. solanacearum*, offering a more environmentally sustainable alternative to chemical pesticides (Kour et al., 2024). These microorganisms are eco-friendly and adaptable to various environmental conditions, including changes in temperature, pH, and stress.

Plant growth-promoting rhizobacteria (PGPR) are a group of microorganisms that promote plant growth by suppressing plant pathogens through both direct and indirect mechanisms (Chandran et al., 2021). PGPR primarily inhabit the soil rhizosphere and initially colonize the plant root system as epiphytes. These bacteria can enter the plant as endophytes and form mutualistic interactions with root exudates through the cortex cells (Ferrante et al., 2023). Once PGPR successfully colonizes the root surface and interior tissues, it may traverse apoplastic barriers (Akhtyamova et al., 2023). Root exudates, which contain organic compounds such as sugars, amino acids, and secondary metabolites, serve as signals that facilitate microbial diversity and attract beneficial microbes (Espinosa-Palomeque et al., 2025). Therefore, PGPR enhances the plant growth directly in the production of nutrient accumulation, phosphate solubilization, nitrogen fixation, and production of phytohormones (Espinosa-Palomeque et al., 2025; Han, 2024). Similarly, the indirect mechanisms suppress the phytopathogens by synthesizing siderophore, hydrogen cyanide, ACC deaminase (Vishwakarma et al., 2020), producing antimicrobial enzymes (chitinase, protease, and lipase) (Chandran et al., 2021; Soliman et al., 2023), and the induction of systemic resistance (ISR) in the host plant (Carlson et al., 2020; Kashyap et al., 2022, 2023; Khan et al., 2020; Walters and Fountaine, 2009). In modern agriculture practice, PGPRs are used as natural biopesticides, biofertilizers, and bioinoculants, offering a sustainable alternative to chemical pesticides (Aloo et al., 2022; Rehman Hakeem et al., 2020). Several PGPR strains, including *Pseudomonads*, *Bacillus* (Attia et al., 2020; Yadav et al., 2023), and *Azotobacter* species, have been isolated for their ability to inhibit phytopathogenic microorganisms and improve plant health (Aloo et al., 2022). For instance, *B. ambifaria* has been shown to degrade fusaric acid metabolites produced by *Fusarium* species, reducing disease symptoms in barley seedlings as reported by Simonetti et al. (2018). Coenye and Vandamme (2003) disclosed that *B. cepacia* has been found to reduce root rot diseases in rice and corn. Siddiqui and Shaukat (2004) employed *P. aeruginosa* 7NSK2 and *P. fluorescens* strain CHA0 to reduce the negative effects of root-knot nematodes on tomatoes through the induction of systemic resistance. Beyond single inoculants, a synergistic combination of arbuscular mycorrhizal fungi (AMF) and PGPR enhances nutrient uptake and plant growth under stress, as reported by Fasusi et al. (2023). The study conducted by Islam et al. (2016) employed the newly identified PGPR strains, *Pseudomonas stutzeri*, *Bacillus subtilis*, *Stenotrophomonas maltophilia*, and *Bacillus amyloliquefaciens* are successfully colonized cucumber roots from seed treatment against *Phytophthora capsica*. In *Vigna radiata*, the strains MRP-7, MRP-8, and MRP-12 showed inhibition of *Macrophomina phaseolina* and enhanced the plant growth and soil health in pot experiments, as proved by Khan et al. (2024). *Bradyrhizobium japonicum* PP236806 and *Bacillus subtilis* PP250150 reduced cotton damping off disease against *Fusarium oxysporum* and *Rhizoctonia solani* (Afify and Ashour, 2024). Similarly, Syed Nabi et al. (2021) reported *Bacillus aryabhattai* SRB02 strain has a strong significant inhibition against tomato wilt disease via inducing phytohormone and amino acids.

Additionally, Mozumder et al. (2022) found that the *P. aeruginosa* A7 has been shown to reduce the survivability of *F. oxysporum* f. sp. *ciceris* in chickpea plants, increasing biomass yield under stress conditions. Although various approaches exist for plant disease management, microorganisms provide a more environmentally sustainable strategy for enhancing plant growth and controlling pathogenic infections. While studies on the synergistic effects of individual PGPR strains have demonstrated significant biocontrol potential, current literature lacks comprehensive investigations into the efficacy of PGPR consortia in improving plant resistance and growth under pathogens stress, including *Fusarium oxysporum* infection. In fact, effective PGPR consortia play a critical role in suppressing diverse pathogens, such as *Septoria protearum*, *Verticillium dahlia*, and *Cercospora canescens*, thereby facilitating plant-microbe interactions that contribute to biocontrol efficacy. Existing research highlights the significant bio-control potential of PGPR strains in these contexts.

The current study aims to investigate how a PGPR consortium influences the suppression of *Fusarium oxysporum* infection in tomato seedlings under controlled greenhouse conditions. In addition to pathogen suppression, the selected strains were evaluated for drought tolerance, biofilm formation, and biosurfactant activity. Their compatibility with fungal and bacterial pathogens was evaluated using *in vitro* culture filtrate assays. The purpose of this study was to assess the efficacy of the PGPR consortium treatment in stimulating tomato growth, improving disease resistance, and creating bioactive compounds with antimicrobial capabilities, which were analyzed using GC-MS. Additionally, gene expression analysis was undertaken to evaluate the defense mechanisms activated by the antagonistic activity of the PGPR strains. A metagenomic profiling was performed to ascertain the soil microbiome during pathogen infection. This study also aimed to investigate the role of PGPR in supporting and maintaining key physiological parameters and antioxidant defense mechanisms, including superoxide dismutase, catalase, phenolic, and flavonoid accumulation, in response to fungal infection. Ultimately, this research seeks to develop safer, more ecologically friendly methods for managing *F. oxysporum* and tomato wilt disease in agricultural contexts.

## 2 Materials and methods

### 2.1 Isolation and bacterial culture preparation

Two bacterial strains, used for this study were isolated from agriculture field soil (VIT University-Vellore, Tamil Nadu, India) using the serial dilution and spread plate technique, followed by quadrant streaking on nutrient agar medium to obtain pure cultures. The strains were initially assessed for PGPR traits, salt tolerance, antibiotic resistance activity, and compatibility (not mentioned in this article). A greenhouse experiment was conducted using individual PGPR strains and their co-culture treatments. For seed biopriming, the bacterial culture was grown overnight in fresh nutrient medium until it reached the logarithmic growth phase. The resulting turbid culture was centrifuged at 5,000 rpm for 10 min, and the pellet was then washed twice with a 0.85% saline (NaCl) solution (Helal et al., 2022). The bacterial suspension was adjusted to an optical density (OD) of 0.6 at 600 nm using the same saline solution.

### 2.2 Collection and maintenance of the isolated strains and pathogens

The strains, *Pseudomonas aeruginosa* and *Burkholderia cepacia* were sub-cultured on nutrient agar medium and stored at 4°C. For long-term storage, the bacterial strains were preserved in 50% (v/v) glycerol at −80°C. Pure culture of fungal pathogens was maintained using appropriate media, including *F. oxysporum* (growth period of 7 days), *R. solanacearum* (growth period of 14 days), *Septoria protearum* (growth period of 10 days), *Verticillium dahlia* (growth period of 7 days), and *Cercospora canescens* (growth period of 7 days) on potato dextrose agar (PDA). This culture was retrieved from the Microbial Type Culture Collection and Gene Bank at the Institute of Microbial Technology, Chandigarh, India. The fungal pathogen *F. oxysporum* was selected for *in vivo* trials.

### 2.3 Evaluation of antagonistic activity assay *in vitro*

#### 2.3.1 Compatibility assay

The antagonistic activity was assessed to evaluate the compatibility between the two bacterial strains: *P. aeruginosa* and *B. cepacia*. This assay was designed to further examine the interactions between the bacterial strains chosen for the study. In the middle of a nutrient agar plate, a single bacterial strain was vertically streaked and then incubated for 24 h at 37°C. After the specified time period, the other strain was streaked perpendicular to the initial strain. The plate was kept at 37°C for an additional 24 h, and any inhibition zones between the strains were observed. Nutrient agar plates with no bacterial inoculation were used as controls (Irabor and Mmbaga, 2017; Prasad and Babu, 2017).

#### 2.3.2 Screening of antibacterial activity

Antibacterial activity of the endophytic bacteria isolates *P. aeruginosa* and *B. cepacia* against *R. solanacearum* was assessed using the agar well diffusion method. *R. solanacearum* was cultured overnight on a CPG broth medium (Casamino, peptone, and glucose) and was standardized to a specific colony-forming unit (10^6^ CFU/mL) concentration. The CPG agar medium was sterilized, poured into plates, and was allowed to solidify. A well of 8 mm diameter was cut at the center of each plate, and *R. solanacearum* was uniformly spread across the plate surface using sterile cotton swabs. Each well was loaded with 200 μL of freshly cultured bacterial suspension (*P. aeruginosa* and *B. cepacia*), and the plates were incubated for 24 h at 37°C. The diameter of the inhibition zones was measured in millimeters (mm) to ascertain antibacterial activity in triplicate. The sterile distilled water served as a control (Cao et al., 2018; Madriz-Ordeñana et al., 2022).

#### 2.3.3 Screening of dual culture assay for antifungal activity

The antagonistic activity of test strains *P. aeruginosa* (VITK-1) and *B. cepacia* (VITK-3) was evaluated against selected plant pathogens: *F. oxysporum*, *S. protearum*, *V. dahlia*, and, *C. canescens*. The fungal pathogens were cultured using appropriate media, prior to the dual culture assay, as described previously in section 2.2.

Bacterial strains were standardized to an optical density OD_600_ = 0.5 (approximately 10^6^ CFU/mL) and streaked 2 cm from one edge of a PDA agar plate, while the fungal culture was streaked 1 cm from the opposite edge on the same medium. The plates were kept at 25°C for 7 days, and the inhibition rate was calculated using Equation 1. The experiment was carried out in three replicates with a completely randomized design.


(1) $$\text{Inhibition rate}\%=\mathrm{RC}-\mathrm{RI}/{\mathrm{RC}}^{*}\;100$$

where, RC is the distance from the edge of the fungal colony on control plates, RI is the distance from the fungal colony to the bacterial inoculum on treated plates.

Using PDA (potato dextrose agar) agar plates and the dual culture method, the antagonistic activity of the bacterial strains against the fungal pathogen was evaluated. In this technique, both the bacterial strains as well as the fungal pathogen was co-inoculated and incubated in the same plate, to observe their corresponding antagonistic activity. The bacterial strains were individually streaked 2 cm from the edge of the petri dish on opposite sides. An agar plug was excised using a cork borer and placed at the center of the plate, where the fungal culture was subsequently introduced. The inhibition was determined using Equation 1, and was compared with the test and control plates (Anith et al., 2021). PDA plates with the fungal culture alone served as the control.

#### 2.3.4 Screening of antagonistic activity of culture filtrate (%)

The antibacterial and antifungal activities of the cell-free supernatants (CFS) from VITK-1 and VITK-3 were tested against various pathogens. Bacterial strains were cultured for 24 h at 37°C in 100 mL of nutrient broth until they reached the stationary phase. Following a 10-min centrifugation at 10,000 rpm, the cultures were passed through a 0.4 μm sterile nylon membrane syringe filter. The resultant filtrate was mixed with PDA agar medium at final concentrations of 25 and 50% (v/v).

Antifungal activity was analyzed using PDA plates incorporated with the aforementioned CFS. These plates were incubated at 25°C after the fungal strain was inoculated in the center of the plates. The progress in growth of fungal mycelium was observed from day 1 to day 7. PDA plates with the fungal culture without CFS alone served as the control.

Antibacterial activity of the CFS was determined using a selective media formulated by mixing CFS with CPG (Casamino, peptone, and glucose) agar at 25 and 50% v/v concentrations. Selected bacterial pathogens were inoculated uniformly over the entire surface of CPG agar plates incorporated with CFS, using a sterile cotton swab following incubation for 48 h at 35°C. The rate of inhibition was recorded and measured using Equation 1. CPG plate with the bacterial pathogen without CFS alone served as the control (Mozumder et al., 2022; Simonetti et al., 2018).

#### 2.3.5 Determination of inhibition zone of culture filtrate against plant pathogens

*In vitro*, an agar well diffusion assay was used to evaluate the inhibition zone. Bacterial isolates were cultured in nutrient broth at 37°C for 24 h. After incubation, the culture filtrate was centrifuged at 10,000 rpm for 10 min. As previously described, 100 μL of the obtained culture filtrate was added to selective media, such as PDA and CPG medium, depending on the type of pathogen. A well, 3 cm from the edge of the Petri dishes, was filled with the culture filtrate. The 7-day-old fungal mycelia and bacterial pathogen were placed at the center of the plate and incubated at 25°C for the specified period. The inhibition zones around the bacterial strain against the fungal and bacterial pathogens were measured using Equation 1. Control plates were inoculated with pathogens, and the wells were filled with sterile distilled water (Ali et al., 2020).

### 2.4 Screening of drought tolerance and biosurfactant and biofilm formation

The bacterial strains (*P. aeruginosa* VITK-1 and *B. cepacia* VITK-3) in LB (Luria Bertani) broth, having different concentrations using different concentrations (0, 100, 150, 200, 500, and 1,000 mg/L) of PEG 4000 (polyethylene glycol; mol. Wt = 4,000) for 24 h (0.6 at OD 600 nm) at 37°C (Muthuraja et al., 2023). The bacterial cultures were incubated under static conditions prior to drought tolerance analysis and under shaking conditions (150 rpm) for determination of biofilm formation. Drought tolerance was evaluated using a spot inoculation method employing LB agar medium, and biofilm production was assessed using the method described by Ali et al. (2020). LB broth inoculated with bacterial strains that are devoid of PEG, served as the control.

Biosurfactant production was screened using the drop-collapsed approach outlined by Habib et al. (2023), with minor modifications. The selected bacterial isolates were inoculated into minimal salt medium (MSM) containing 0.5 g NH_4_Cl, 4 g NaCl, 0.5 g KH_2_PO_4_, 1 g NA_2_HPO_4_, 0.5 g MgSO_4_.7H_2_O, 1 L dH_2_O at pH 7.0 and incubated for 24 h at 37°C. After incubation, cultures were centrifuged for 10 min at 6,000 rpm. By mixing 3 mL of the supernatant with 2 mL of mineral oil, vortexing for 2 min, and then incubating for 12 h, the emulsification index was calculated. Equation 2 was used to determine the emulsification index:


(2) $$\begin{array}{l} E24\;\text{index}\;(\%)\\ =\text{Height of emulsion collapsed}/\text{total height}\;{\text{of solution}}^{*}\;100 \end{array}$$

Biofilm formation was screened using the test tube method. In a test tube containing LB broth (1 mL in 10 mL), bacterial isolates were cultured and incubated for 24 h at 37°C. After incubation, the test tubes were washed thrice with sterile distilled water and stained with crystal violet for 20 min. The tubes were then washed with sterile water to remove any excess strain, and the presence of biofilm formation was observed (Ali et al., 2020).

### 2.5 Primary screening of bioactive compounds using GC-MS analysis

The qualitative analysis for *P. aeruginosa* (VITK-1) and *B. cepacia* (VITK-3) was performed using GC-MS (gas chromatography-mass spectrometry). The purpose of the analysis was to find bioactive compounds that the bacterial strains produced that might prove effective against phytopathogens. Sample preparation was done by inoculating the strains in nutrient broth medium for 48 h at 30°C (150 rpm). To extract the cell-free supernatant, the cultures were centrifuged for 10 min at 1,000 rpm following incubation. The supernatant was then extracted by mixing with an organic solvent (n-hexane or ethyl acetate in a 1:1 ratio) twice and incubated overnight with shaking. A separating funnel was utilized to split the mixture into layers after extraction. A rotary evaporator was used to separate and concentrate the top layers. Once the solvent had evaporated, the extract was dried completely, weighed, and dissolved with HPLC-grade methanol (Sigma Aldrich) for GC-MS analysis (Heo et al., 2022). To identify bioactive compounds with potential phytopathogenic activity, the analysis was outsourced to TUV SUD South Asia Pvt. Ltd. (Ranipet, Tamil Nadu, India), following established standardized procedures.

### 2.6 Seed priming with bacterial suspension treatment

As indicated in Table 1, two PGPR strains and their consortia, comprising a total of eight treatment groups, were used in the greenhouse experiment. Commercially purchased tomato seeds (variety PKM1) were surface sterilized by washing twice with 72% ethanol, followed by treatment with sodium hypochlorite for 5 min, and rinsed with sterile distilled water. After that, seeds were left to soak for an hour in the bacterial suspension (described in section 2.1). One-month-old tomato seedlings were employed in the *in vivo* experiment, which was conducted in triplicates using a completely randomized block design. After being removed from the pro tray, the seedlings were washed with water and immersed in 30 mL bacterial suspension for 30 min. The control seedlings were submerged with sterile distilled water. After the treatment, the seedlings were transferred into separate pots containing a soil mixture composition of red soil, sand, vermicompost, and cocopeat in a 1:1:1:1 ratio, along with the respective bacterial suspension. The pots were maintained in a greenhouse at a controlled temperature of 25°C and 30°C with relative humidity ranging from 55 to 65% at the VIT School of Agricultural Innovations and Advanced Learning (VAIAL), VIT, Vellore, India. Ten days post-transplantation, the treated bacterial suspension was reapplied. No additional fertilizer was added for the remainder of the experiment. Three-week-old seedlings were then inoculated with *F. oxysporum* by dipping the seedlings in the pathogen solution for 30 min, after which they were placed back into the pot. Physiological parameters and biochemical characterization analysis were measured 15 days post-inoculation to assess early plant responses to *F. oxysporum*. This period of measurement was selected to show the peak infection stage. Disease severity was measured 6 weeks post-inoculation, and plants were maintained in greenhouse conditions as described.

**Table 1:** Treatment groups for greenhouse experiment.

Treatments	Treatment combination
T1	Control (non-inoculated); no bacterial suspension, no pathogen
T2	*Fusarium* control (no bacterial suspension)
T3	VITK-1 (*P. aeruginosa*; Accession ID: OP102696)
T4	VITK-1 + P (*P. aeruginosa* + *F. oxysporum*)
T5	VITK-3 (*B. cepacia*; Accession ID: PP897814)
T6	VITK-3 + P (*B. cepacia* + *F. oxysporum*)
T7	VITK-1 + VITK-3 (*P. aeruginosa* + *B. cepacia*)
T8	Consortia treatment (*P. aeruginosa* + *B. cepacia* + *F. oxysporum*)

At the final stage of the experiment, plants were uprooted and rinsed with water to measure a number of growth characteristics, including fresh weight, plant height, dry weight, root length, shoot diameter, root fresh weight, chlorophyll content (measured using a SPAD meter), and root dry weight. Finally, samples were dried in a hot air oven at 60°C for 3 days to analyze the biochemical and physiological characteristics for further studies.

### 2.7 Soil characterization

Soil samples were analyzed and characterized by the National Agro Foundations (NAF, Chennai) to better understand the chemical properties, pH, electrical conductivity, and nutrient availability of macronutrients and micronutrients. Characterization of soil is generally performed before the transplantation of seedlings from pro tray to pot (Cottenie, 1980).

### 2.8 Determination of disease resistance (%)

Disease severity was assessed 6 weeks after fungal spore suspension inoculation. Symptoms were evaluated based on the percentage of affected leaves, with a focus on the leaves showing disease symptoms. The disease severity was rated using a 5-point scale: (0) indicated absence of any diseases, (1) indicated that 20% leaves area was affected (lower leaves yellowing), (2) indicated that 20–41% leaves were affected (slightly yellowish), (3) indicated that almost 41–60% leaves were affected (modest wilted), (4) indicated 60–85% leaves were affected (full wilted) and (5) indicated that above 85% leaves were affected (complete plant death). The disease severity index (DSI) was calculated using the following formula (Soliman et al., 2023) (Equation 3).


(3) $$\mathrm{DSI}=\varepsilon \;(\text{Average plant rating})/{\left(\text{Total plants}\times \text{Highest rating}\right)}^{*}\;100$$

### 2.9 Determination of chlorophyll content

Fresh leaf samples (0.2 g) were obtained and homogenized with 80% chilled acetone, following the protocol described by Rajalakshmi and Banu (2015) with slight modification. At 4°C, the homogenized samples were centrifuged for 10 min at 5,000 rpm. After transferring the supernatant to a test tube, acetone was used as the blank, and a UV–visible spectrophotometer was used to determine the absorbance at wavelengths of 663 nm and 645 nm.

### 2.10 Estimation of total protein content

Total protein content was estimated using Lowry’s method with slight modifications. Protein was extracted from 0.1 g (100 mg) of fresh leaf samples using PBS buffer at pH 7 and vortexed. A microplate reader was used to detect the samples’ fluorescence at 660 nm. Fifty milliliters of distilled water was used to dissolve 50 mg of Bovine serum albumin (BSA) to create a stock solution. A graph was then constructed to calculate the concentration of the unknown sample (Sarkar et al., 2020).

### 2.11 Plant extraction and determination of antioxidant activity

Plant samples (0.4 g) were dried at 60°C for 2 days. In a conical flask, 10 mL of methanol (HPLC grade) were combined with dried samples. The flask was covered with aluminum foil to prevent evaporation, and the mixture was vortexed overnight. The sonication technique was applied to enhance agitation, and the mixture was then filtered through Whatman No. 1 filter paper. For additional analysis, the extracted sample was dried, resuspended in methanol to form a stock solution, and stored at −20°C.

Antioxidant activity was evaluated using the DPPH (1,1, diphenyl-2picrylhydrazyl) assay with slight modifications (Phuyal et al., 2020). The concentration of the plant extract was diluted with methanol to a range of 20 mg/L–100 mg/L. A standard L-ascorbic acid stock solution was used for comparison. The different concentrations of plant extract were mixed with a 0.004% DPPH solution (dissolved in methanol) and incubated for 30 min at room temperature. Methanol and DPPH were used as blank and control, respectively. Following incubation, the absorbance was measured at 517 nm, and the antioxidant activity was computed using the specified formula (Supplementary Data S1).

### 2.12 Superoxide dismutase enzyme activity

Superoxide dismutase (SOD) activity was determined following the methods described by Venkat et al. (2023), with minor modifications. To mention briefly, fresh leaf samples (0.2 g) were homogenized using a pre-cooled mortar and pestle with 2 mL of extraction reaction buffer containing 0.5 M phosphate buffer (pH 7.5), 1 mM EDTA, 1% PVP, and Triton X-100. The resulting mixture was transferred to the tubes and were centrifuged at 15,000 rpm at 4°C for 10 min. The supernatant was carefully collected separately. Next, 0.1 mL of the supernatant was mixed with a reaction buffer containing 1.5 mL of 0.1 M phosphate buffer (pH 7.8), 200 mM methionine, 0.1 M sodium carbonate (Na_2_CO_3_), an equal volume of 2.25 mM nitro blue tetrazolium, 60 mM riboflavin, 3 mM EDTA, and distilled water, respectively. The assay mixture was incubated under a 15 W fluorescent lamp and in the dark condition for 10 min. The reaction was carried out under both light and dark conditions, excluding the enzyme extract. Absorbance was measured at 560 nm using a UV–visible spectrophotometer. Enzyme activity, measured in units of enzyme activity mg^−1^ protein h^−1^, was determined from the percentage change in color.

### 2.13 Catalase activity

Catalase enzyme activity was measured using the method described by Venkat et al. (2023). A.0.2 g sample of fresh leaf tissue was homogenized using a pestle and mortar. After adding 2 mL extraction buffer (as described above), the mixture was centrifuged for 20 min at 4°C at 10,000 rpm. After collecting 200 μL of the supernatant, 600 μL of reaction buffer containing 0.1 M KH_2_PO_4_ and K_2_HPO_4_ (pH 7.3) was added. Finally, 200 μL of H_2_O_2_ was then added, making a total volume of 1 mL. At 240 nm, absorbance was measured (*E* = 39.4 mM^−1^ cm^−1^).

### 2.14 Phyto compound production

Two techniques were employed to determine the phenolic and flavonoid content using the Folin–Ciocalteu and aluminum chloride methods, respectively. For phenolic content, 100 microliters of the extracted plant sample (described in section 2.11) was mixed with 400 microliters of methanol, followed by the addition of 150 microliters of Folin Ciocalteu reagent and 20% sodium carbonate (Chavan et al., 2013). The solution was vortexed thoroughly and incubated in the dark for 1 h. Methanol and Folin–Ciocalteu reagents were used as blanks. A standard curve was plotted using gallic acid to determine the total phenolic content in the test sample, and absorbance was measured at 650 nm. For flavonoid content, 100 microliters of the test sample was combined with 400 microliters of methanol, followed by the addition of 100 microliters of 10% AlCl_3_ and 1 M sodium acetate (Phuyal et al., 2020; Rakesh et al., 2021). After vortexing, the mixture was allowed to sit at room temperature for 45 min. Using a microtiter plate with methanol alone as the blank (without test samples), absorbance was measured at 415 nm.

### 2.15 Electrolyte leakage

To measure the electrolyte leakage, 200 mg of fresh leaf samples were washed with distilled water and cut into small pieces. The samples were put in a test tube with 20 mL of sterile distilled water, which was covered with aluminum foil to prevent electrolyte leakage. Initial measurements were recorded after 24 h of incubation. After that, the test tubes were submerged in the water bath set at 100°C for 1 h. The final electrical conductivity (EC) was measured following cooling. The percentage of EC was calculated based on the initial and final EC values using the following Equation 4 (Hayat et al., 2008; Slabbert and Krüger, 2014).


(4) $$\%\mathrm{EC}={\left(\mathrm{EC}1/\mathrm{EC2}\right)}^{*}\;100$$

### 2.16 Quantification using RT-PCR

The expression of pathogen-defense-related genes in *S. lycopersicum* was analyzed using quantitative real-time PCR (qRT-PCR). Gene expression was assessed in fungal-infected plants treated with individual bacterial strains and their consortia. After 15 days of infection, root samples were collected from eight treatment groups and a control group, with biological triplicates for each. The samples were immediately frozen in liquid nitrogen (N_2_) and stored at −80°C. RNA extraction was carried out using RNA-Isoplus (Takara) with 200 mg of fresh leaf tissue from each treatment group. The RNA yield was quantified using a Nanodrop spectrophotometer (Thermo Scientific, United States). Reverse transcription was carried out using a cDNA synthesis kit (Himedia) to convert the RNA into complementary DNA (cDNA), with nuclease-free water. Real-time PCR amplification was performed using a SYBER Green master mix (Takara) on a Bio-Rad thermal cycler (United States). Briefly, the amplification reaction mixture contained 2 μL of both forward and reverse primers followed by adding 2 μL of nuclease-free water, 5 μL of the reaction (SYBER) mixture, and 1 μL of cDNA template with a total volume of 10 μL. Before setting the qRT-PCR conditions, gradient PCR was amplified using a cDNA template and varying primer annealing temperatures (ranging from 55°C to 62°C) with a melting curve stage of 95°C for 10s and 60°C for 1 min. The optimized qRT-PCR conditions were as follows: an initial denaturation at 95°C for 10 min, followed by 40 cycles of 95°C for 15 s, 56°C for 10s, and 72°C for 15 s. Primers were designed using Oligo 7 software (Supplementary Table S2) based on the *S. lycopersicum* gene sequence obtained from BLAST and NCBI. The actin gene was used as the reference gene for *S. lycopersicum* (Arakkal Thaiparambil and Radhakrishnan, 2023).

### 2.17 Metagenomic profiling of rhizosphere soil

Soil samples were collected from the rhizosphere of tomato plants by gently removing the soil from around the plant roots. Treatment samples (T1, T2, T5, and T8) were selected for metagenomic analysis and was labeled as: VITAKM1, VITAKM2, VITAKM3, and VITAKM4. Crude genomic DNA from each treatment group was extracted using the Xploregen Kit according to the manufacturer’s instructions. The purity and quantity of the nucleic acids were assessed using a NanoDrop spectrophotometer (Thermo Scientific, United States) and agarose gel electrophoresis (Bio-Rad Horizontal Unit). Illumina barcoded adapters were used to generate DNA libraries, which were then purified with Ampure beads and measured with a Qubit dsDNA high-sensitivity assay kit. Using a 2×300PE V3–V4 sequencing kit, the sequences were carried out using an Illumina Miseq. The following accession codes are given for the raw sequencing data, which was submitted to the Sequence Read Archive (SRA) databases and National Center for Biotechnology Information (NCBI) database: SAMN47269860, SAMN47269861, SAMN47269862, SAMN47269863, and Bio project accession ID were retrieved as PRJNA1233473.

### 2.18 Bioinformatics analysis

The raw data quality was assessed using FastQC and MultiQC to evaluate the data generation process, followed by adaptor trimming. The trimmed reads were then processed using steps including merging pair ends, performing a chimeric check (removing 10% of chimeric sequences), and estimating OTU abundance, all within the QIIME workflow. These steps ensured high accuracy in the investigation, which included analysis at both the phylum and genus levels. Additionally, metagenomic data analysis was performed to assess alpha and beta diversity. The National Center for Biotechnology Information (NCBI) submitted the processed data for further reference and accessibility (Botlagunta and Babu, 2024; Skipper et al., 2022).

Alpha diversity was measured using the alpha tool, with input data filtered to include indices such as Shannon (measures both species richness and evenness), Chao1 (species richness), and Simpson (relative abundance). The abundance of species was measured within a sample and compared with the diversity across different samples. The Chao1 index estimates species richness, including unobserved species. Shannon’s index reflects the uncertainty in the distribution of individuals among species within the community. The Simpsons index estimates the relative abundance of each species within the community. Based on these calculations, the variance in biodiversity was assessed for each treatment group, with a significance level of *p* < 0.05.

Using a Bray–Curtis dissimilarity matrix and principal coordinates analysis (PCoA), beta diversity was assessed. The visualization of the sample was represented on the x-axis and y-axis, with each axis corresponding to the primary dimensions of variance between the samples. These axes describe the largest and second-largest sources of variation. Permutational ANOVA was used to evaluate the plot variations’ statistical significance. In order to estimate the correlation distance between the genera, the heat maps were also created using R and QIIME. Heat maps incorporated data on rarefaction curves, species prevalence, and microbial metabolism. The relative abundance of genera was considered the key distinguishing feature between samples.

### 2.19 Statistical analysis

All the tests and experiments mentioned in the current study was performed in triplicates and the results are expressed as mean ± standard deviation (SD), calculated using Microsoft Excel. Graphical data was generated using GraphPad Prism and JMP Pro 18, and Duncan’s multiple range test was also performed. The experimental design adopted here followed a completely randomized design. Significant differences among treatments were calculated using one-way ANOVA followed by a two-tailed Student’s *t*-test and Duncan’s multiple range test for comparisons.

## 3 Results

### 3.1 Isolation, identification, and characterization of bacterial strains

**Figure 1:**
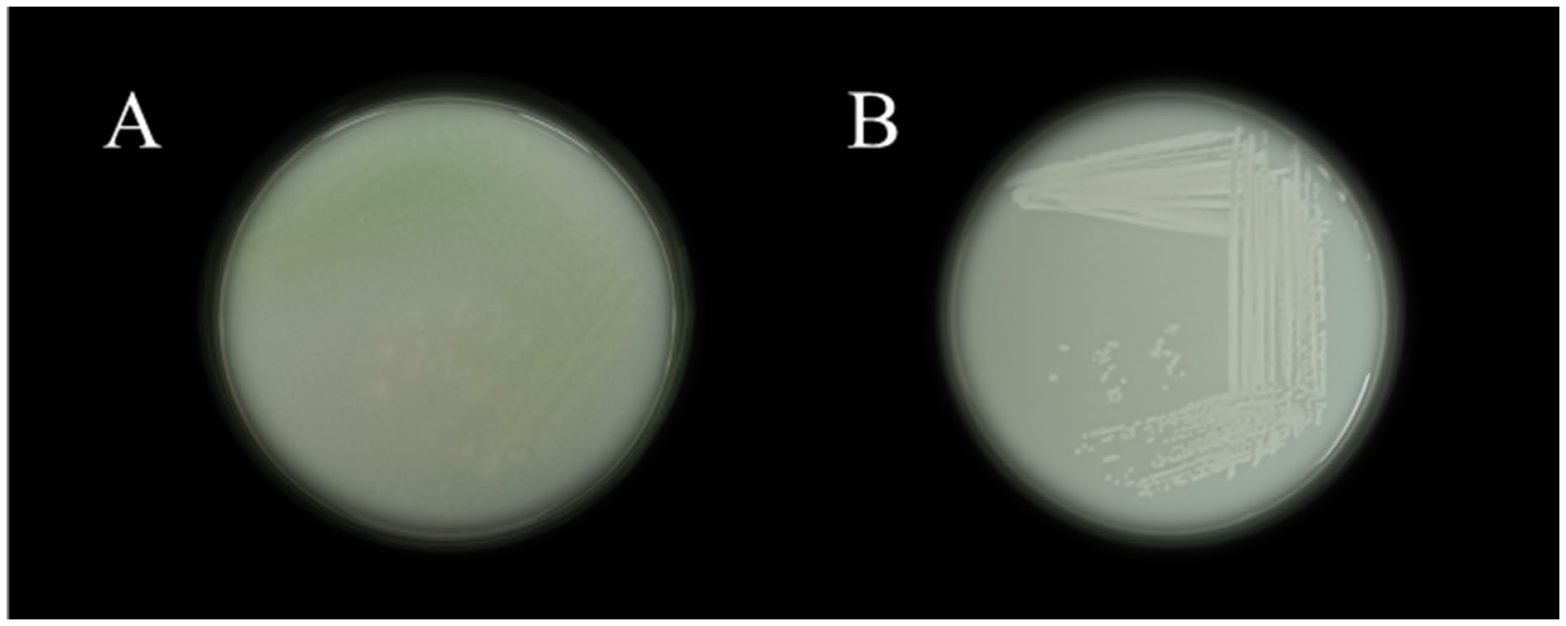
The isolated colonies on nutrient plates VITK-1 **(A)**, and VITK-3 **(B)**.

The isolated bacterial colonies on Nutrient agar plates upon quadrant streak is displayed in Figure 1A VITK-1 and Figure 1B VITK-3. Detailed colony characterization and morphological analysis, revealed that VITK-1 appeared to be dull-white in color with round, entire margin, and slight elevation. The colonies were opaque in nature, had a notable bluish-green tint in the vicinities of streak lines, and were small to medium-sized. Whereas, colonies of the strain VITK-3 has similar size as compared to VITK-1, but was white/creamy in color round marginated and non-transparent. Microscopic examination revealed that, the strains appeared to be rod/coccoid shape and were Gram-negative. From 16 s rRNA sequencing and BLAST identification, VITK-1 was identified to be *P. aeruginosa* with a similarity index of 99%, and the strain VITK-3 was identified to be *B. cepacia* with a similarity index of 99.5%. The GenBank accession numbers assigned to these sequences were SUB11876418 and SUB14520450, respectively.

### 3.2 Antagonistic activity

Table 2, which shows the growth inhibition patterns of *P. aeruginosa* and *B. cepacia*, illustrates the findings of the antagonistic activity analysis. The extent of inhibition observed reflects the level of compatibility or antagonism between the isolates. Based on the findings, *P. aeruginosa* and *B. cepacia* successfully coexisted on the same nutrient agar plate without inhibiting each other’s growth after a 24-h incubation period. The absence of antagonistic interactions confirms a positive compatibility result, as summarized in the table.

**Table 2:** Antagonistic activity of *F. oxysporum*, +++ powerful, ++ moderate produces, + less produce, and − no production; antagonistic activity of *R. solanacearum* +++ less inhibition, and ++ moderate inhibition; for combination assay of bacterial isolates + combability.

Isolate name	Antagonistic activity (biofilm) (*F. oxysporum*)						Antagonistic activity *R. solanacearum* (CFS)		Compatibility assay	
	0	Fus	VITK-1	VITK-1 + Fus	VITK-3	VITK-3 + Fus	25%	50%	VITK-3	VITK-1
*P. aeruginosa*	−	+++	++	+			++	++	+	
*B. cepacia*	−	+++			++	++	++	++		+

### 3.3 Antagonistic activity of isolated strains

**Figure 2:**
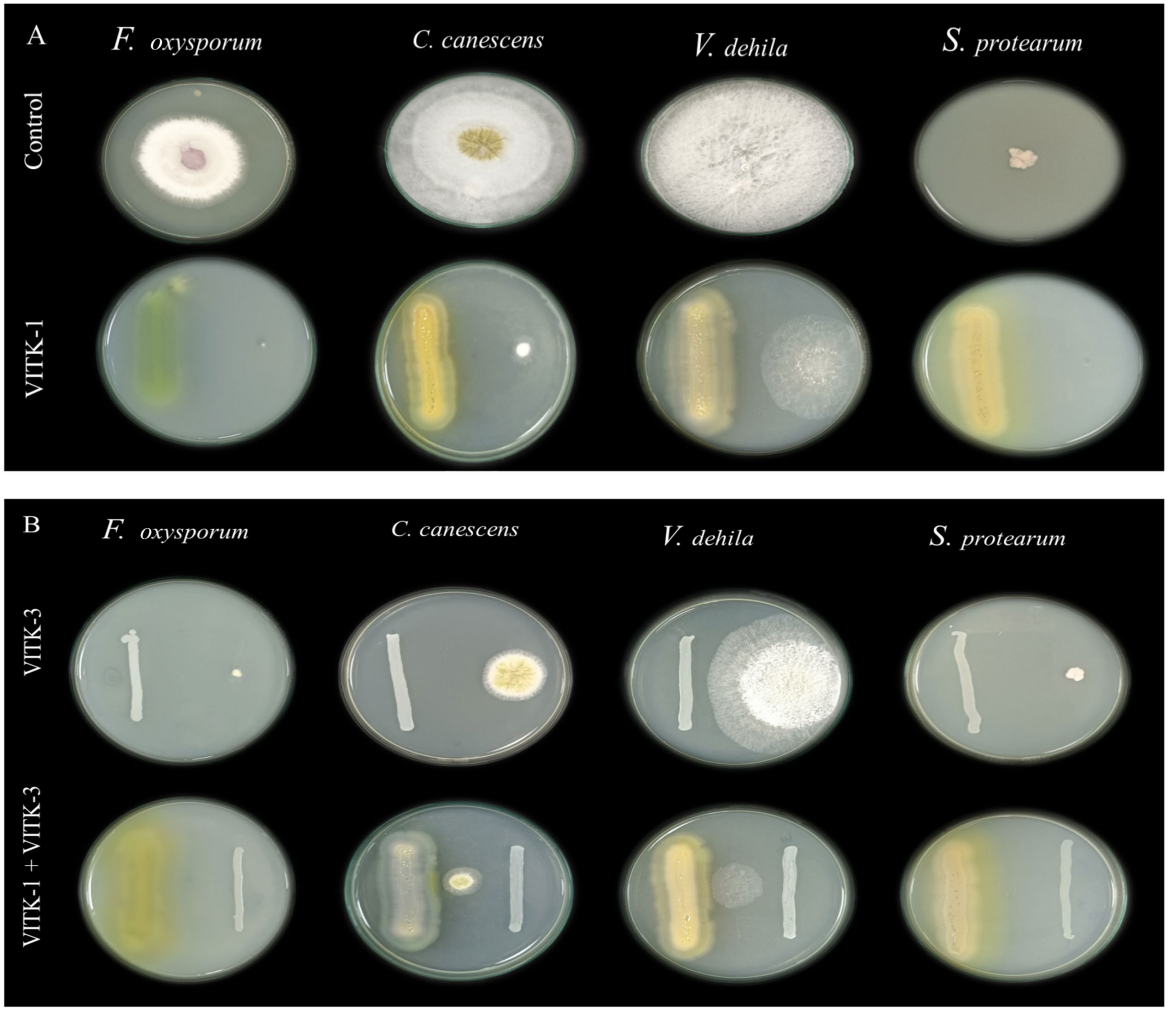
Screening of antagonistic activity assay of *P. aeruginosa* (VITK-1) and *B. cepacia* (VITK-3) against phytopathogens of fungi and bacteria *in vitro*. On dual culture assay, the potential activity of control (in the absence of isolates), VITK-1, VITK-3, and their combination against *F. oxysporum*, *C. canascens*, *V. dahlia*, and *S. protearum* **(A,B)**, after 7 days of inhibition.

**Figure 3:**
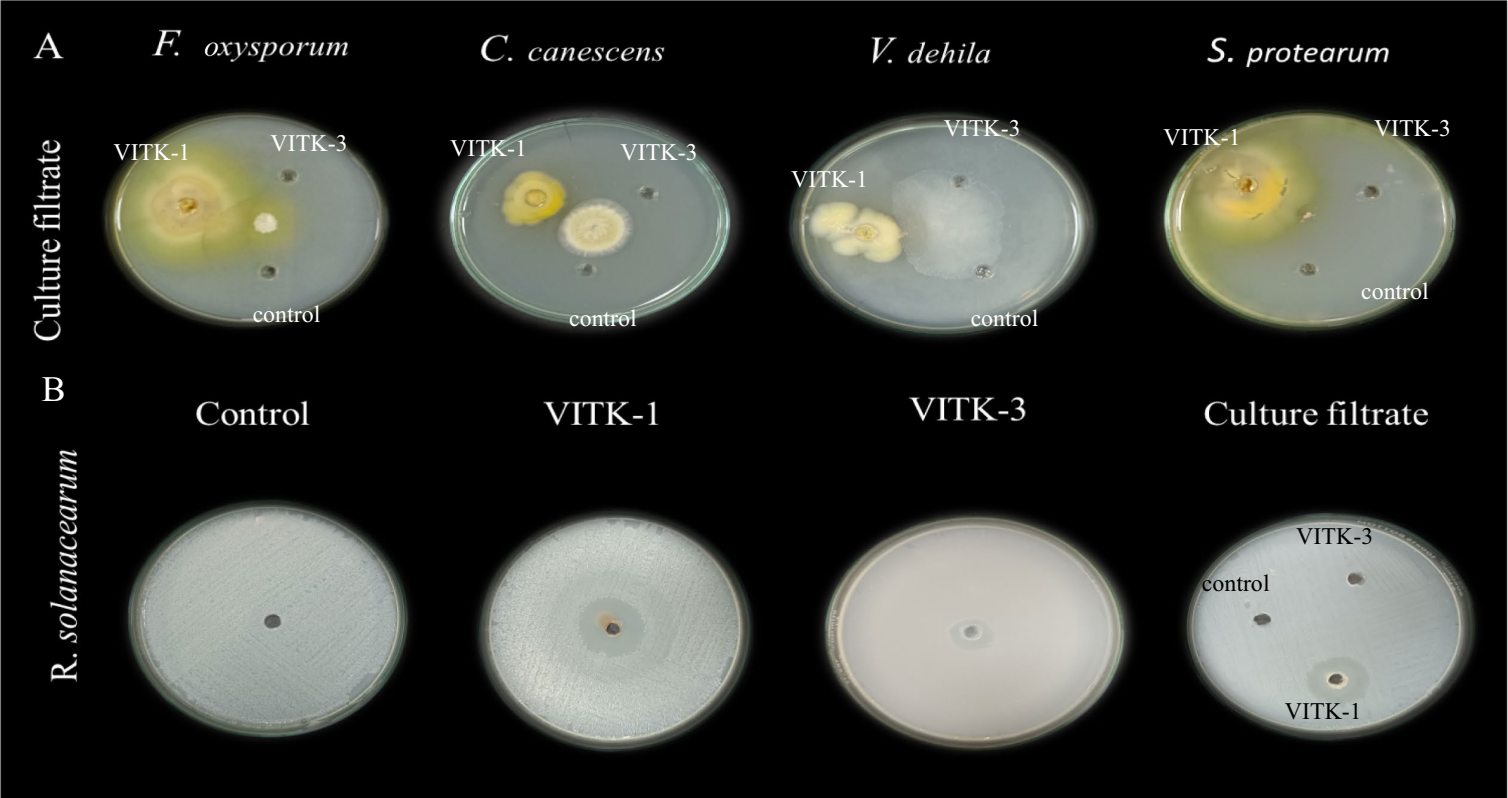
Determination of agar well diffusion assay of antagonistic activity against plant pathogens. **(A)** Culture filtrates (100 μL) of *P. aeruginosa* (VITK-1), *B. cepacia* (VITK-3), and control (sterile distilled water) were loaded into 3 cm wells on PDA plates inoculated with *F. oxysporum*, *C. canascens*, *V. dahlia*, and *S. protearum*. **(B)** The first three plates were loaded with freshly cultured bacterial suspension (200 μL) of VITK-1 and VITK-3 into 8 mm wells on CPG agar against *R. solanacearum*. The fourth plate shows the combined effect of VITK-1 and VITK-3 culture filtrates, along with the control (sterile distilled water), tested against *R. solanacearum* and incubated for 24 h.

**Figure 4:**
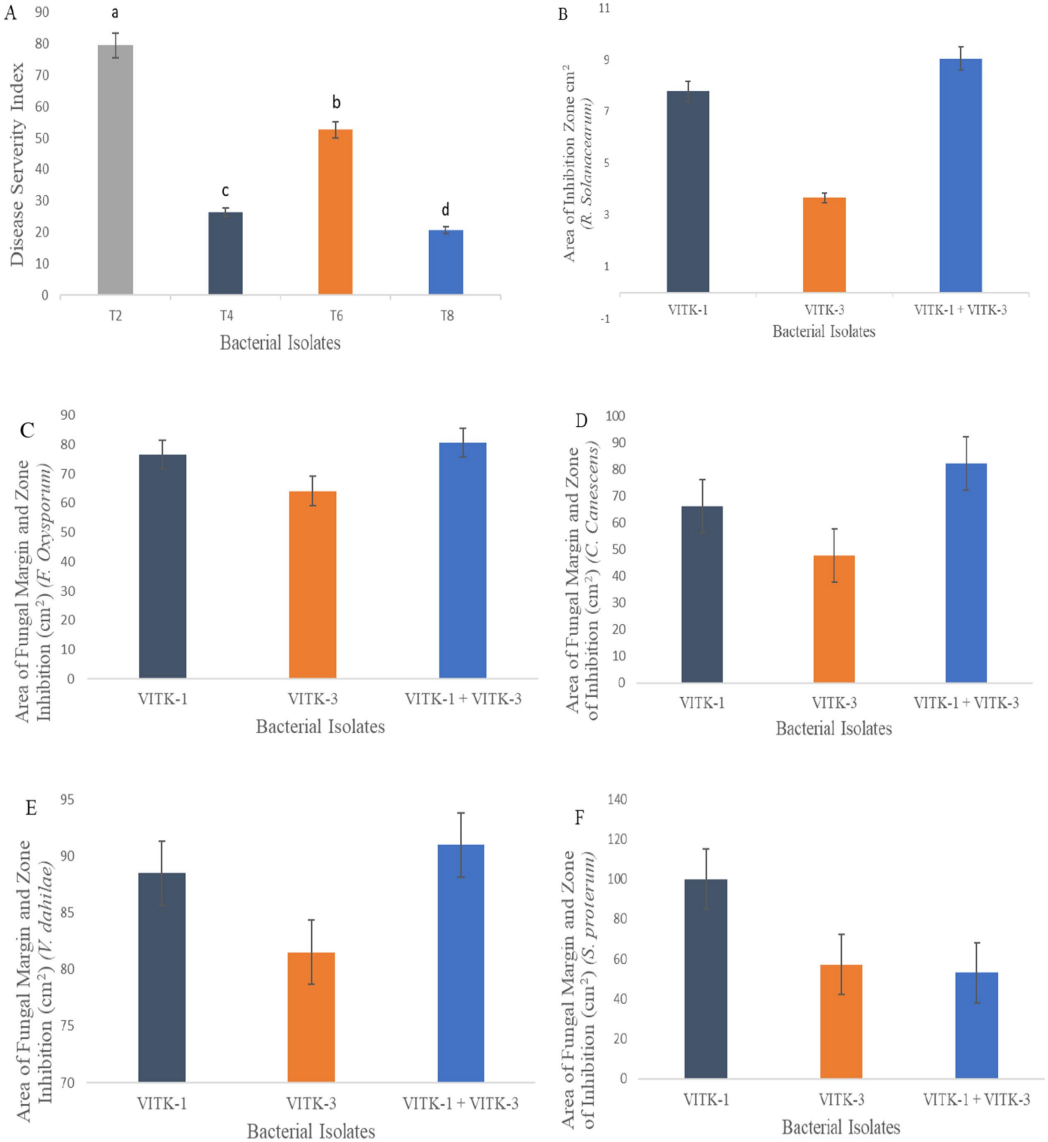
Antagonistic activity assay of VITK-1, VITK-3 and their combinations against plant fungal and bacterial pathogens. Disease severity index against *F. oxysporum* in greenhouse trials **(A)** under experimental conditions (*n* = 3). Area of inhibition rate against *R. solanacearum* **(B)** on CPG growth media. Area of fungal inhibition activity of isolates strains **(C–F)** against all different plant pathogens on dual culture assay.

In the dual culture assay, the strains VITK-1, and VITK-3, and their combination inhibited the growth of test pathogens, including bacterial and fungal pathogens. These included *R. solanacearum* followed by *F. oxysporum*, *S. protearum*, *V. dahlia*, and *C. canescens* was confirmed through the observations (Figures 2A,B, 3A). Quantitative measurements revealed that VITK-1 exhibited the highest growth inhibition rate against *F. oxysporum* (76.5%) in PDA growth media, followed by *C. canescens* (66.1%), *V. dahlia* (88.5%), *S. protearum* (99%), and (71.8%) *R. solanacearum* (Figure 4).

Similarly, the bacterium VITK-3 showed inhibition of mycelia growth and growth suppression of the Gram-negative bacterium *R. solanacearum* at 35.7%, demonstrating antagonistic activity against all tested fungal pathogens. The highest inhibition rate was observed against *V. dahlia* at 81%, while the lowest was observed against *F. oxysporum* at 64.1%, *C. canescens* (47.7%), and *S. protearum* (57.2%) when compared to VITK-1 and the combination of isolates. The combinations of VITK-1 and VITK-3 showed clear inhibition against pathogens in the following order: *V. dahlia* > *C. canescens* > *F. oxysporum* > *R. solanacearum* and *S. protearum* as observed in Figure 4. These results suggest that the combinations of isolates have a synergistic effect in pathogens suppression and is considered compatible for further experiments.

### 3.4 *In vitro* antagonistic activity assay of culture filtrate

**Figure 5:**
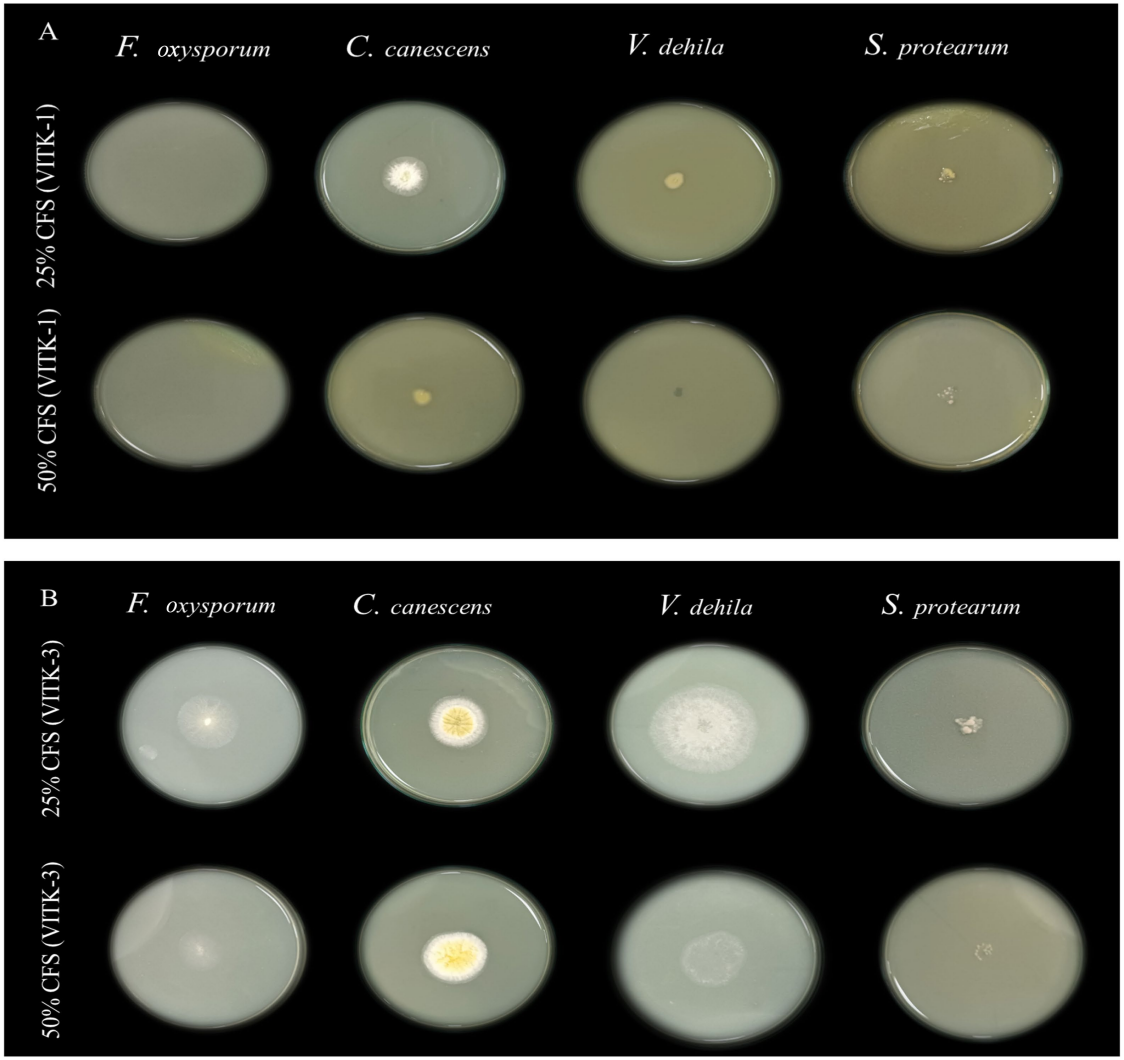
Antagonistic effect of culture filtrate (25 and 50%) of *P. aeruginosa* (VITK-1) and *B. cepacia* (VITK-3) mixed with PDA medium **(A,B)**. The inhibition assays were performed against plant pathogens *F. oxysporum*, *C. canascens*, *V. dahlia*, and *S. protearum*, after 7 days of incubation.

**Figure 6:**
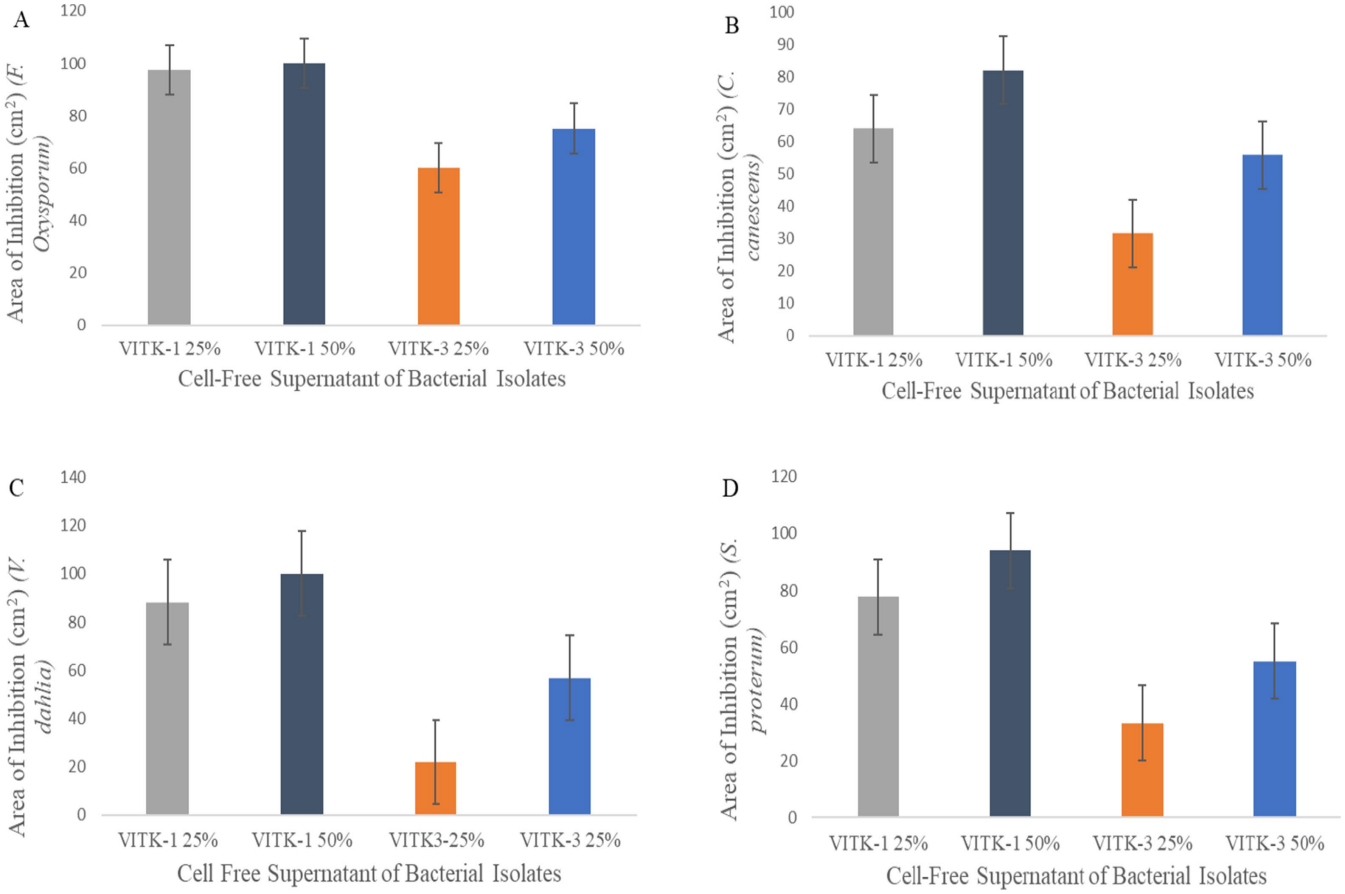
Antagonistic activity of bioactive compounds produced by *P. aeruginosa* and *B. cepacia* against plant pathogens. The culture filtrate was mixed with PDA growth media for the plate assay. Area of fungal inhibition on *F. oxysporum* **(A)**, *C. canescens* **(B)**, *V. dahlia* **(C)**, and *S. proterum* **(D)** obtained after 7 days of inhibition.

The antagonistic activity of VITK-1 and VITK-3 demonstrated significant differences in their ability to inhibit pathogens, likely due to the production of bioactive compounds. The percentage of fungal and bacterial inhibition ranged from 25 to 50% with the culture filtrates. VITK-1 exhibited the highest fungal inhibition against *F. oxysporum*, reaching 97.5%, followed by *V. dahlia* at 88.2%, *C. canescens* at 82%, and *S. protearum* at 94%, as shown in Figures 5A,B, 6. Minimal inhibition of *R. solanacearum* was observed with both VITK-1 and VITK-3, as presented in Table 2. VITK-3 showed a range of inhibition, with the highest inhibition observed for *F. oxysporum* at 75%, and moderate inhibition against other fungal pathogens, including *S. protearum* at 55%, *V. dahlia* at 56.9% and, *C. canascens* at 55.8%. In the agar well diffusion method, 100 μL of VITK-1 and VITK-3 culture filtrate suppressed *R. solanacearum* growth, with inhibition zones measuring 48 ± 1.5 mm for VITK-1 and 14.9 ± 0.05 mm for VITK-3, as shown in Figure 3A. A significant difference in growth inhibition was observed between the pathogens when compared to the control (sterile distilled water) as illustrated in Figure 3A and Table 3.

**Table 3:** Effect of growth inhibition of *P. aeruginosa* and *B. cepacia* against the phytopathogens in agar diffusion method assays by producing bioactive compounds, “−” no inhibition effect against fungi and bacteria.

Isolate name	Agar diffusion assay (*R. solanacearum n* = 3) (mm)	*F. oxysporum*	*C. canescens*	*V. dehila*	*S. protearum*
*P. aeruginosa*	48 ± 1.5	81.4 ± 1.69	89.9 ± 0.95	90.2 ± 1.3	88.3 ± 2.08
*B. cepacia*	14.9 ± 0.05	65.8 ± 0.8	53.8 ± 0.8	—	9.66 ± 0.5

### 3.5 Determination of drought tolerance, emulsification index, and biofilm production of isolated strains

From the results presented in Table 4, the fresh culture of *P. aeruginosa* and *B. cepacia* exhibited the highest growth range at 4,000 mg/L PEG, compared to the control. Drought stress had variable effects, highlighting the importance of understanding and managing osmotic stress. Notably, VITK-1 and VITK-3 demonstrated maximum growth at 1,000 mg/L on LB growth medium after a 24-h incubation period. These results indicate a high level of drought tolerance, with growth responses, categorized as powerful and moderate, as represented in Table 4. Our findings also revealed biosurfactant activities, with VITK-1 producing an emulsification index of 68% while VITK-3 achieved the highest emulsification index of 80% on minimal growth media (Table 4).

**Table 4:** Effect of PGPR features on inoculation of *P. aeruginosa* and *B. cepacia*, +++ powerful, ++ moderate produces, + less produces, and − no production.

Isolate name	Drought tolerance (PEG 4,000 mg/L)						Drought tolerance (biofilm)						Biosurfactant
	0	100	150	200	500	1000	0	100	150	200	500	1000	
*P. aeruginosa*	−	+++	+++	+++	+++	+++	−	+++	+++	+++	+++	+++	++
*B. cepacia*	−	+++	+++	+++	+++	+++	−	+++	+++	+++	+	+	+++

The selected isolated strains were tested for their biofilm-forming ability under biotic and abiotic stress conditions using a test tube assay. *B. cepacia* showed significantly higher biofilm formation compared to *P. aeruginosa* and the control when co-inoculated with fungal mycelial culture after 7 days of incubation period. The biofilm formation levels were categorized using distinct symbols representing strong, moderate, low, and no production, as summarized in Table 2.

### 3.6 Gas chromatography-mass spectrometry analysis

Hexane and ethyl acetate were used to analyze and characterize the bioactive compound production in the culture filtrate of *P. aeruginosa* and *B. cepacia*. A total of 34 compounds were identified from the TUV SUD South Asia Pvt. Ltd. library collection. These compounds were chosen on the basis of their molecular weight, molecular formula, and retention time (RT). Key compounds identified include 4H-Cylcopropal (RT 26)2,2,4-Trimethyl-3-(3,8,12,16-tetramethyl-heptadeca-3,7,11,15-tetraenyl)-cyclohexanol (RT 25), Hexadecane and Pyrrolo [1,2-a] pyrazine-14 (RT 22 and 23), and octadecanoic acid and 2-3,-dihydroxypropyl ester (RT 21). Additional chemical compounds are listed in Table 5.

**Table 5:** List of bioactive compounds produced by *P. aeruginosa* and *B. cepacia* on GC-MS analysis using hexane and ethyl acetate.

Retention time	Compound	MF	MM	Organism
24	Cyclopental	C_5_H_10_	70	*P. aeruginosa*
	Ethyl iso-allocholate	C_26_H_44_O_5_	436	
	Hexadecanoic acid, 1,3-propanediyl ester	C_35_H_68_O	552	
	1,4-Benzendicarboxylic acid, bis(2-ethylhexyl) ester	C_24_H_38_O_4_	390	
12	2-Decenoic acid	C_10_H_18_O_2_	170	*P. aeruginosa*
23	Octadecane, 3-ethyl-5-(2-ethylbutyl)	C_26_H_54_	366	*P. aeruginosa*
	Oleic acid, 3-(octadecyloxy)propyl ester	C_39_H_76_O_3_	593	
	2,4,6,8,10-Tetradecapentaenoic acid	C_36_H_46_O_8_	606	
	3-Methylcyclopentadecylcarbamic acid, t-butyl ester	C_11_H_22_N_2_O_2_	214	
26	17-Pentatriacontene	C_35_H_70_	490	*P. aeruginosa*
	4H-Cyclopropal[5′,6′] benz[1′,2′: 7,8] azuleno [5,6-b] oxiren-4-one, 8,81-bis(acetyloxy)-2a-[(acetyloxy)methyl]-1,2,3,6,8,8-dodecahydro-3, 3a, 6b-trihydroxy-1,1,5,7-tetramethyl	C_26_H_34_O_11_	522	
25	2,2,4-Trimethyl-3-(3,8,12,16-tetramethyl-heptadeca-3,7,11,15-tetraenyl)-cyclohexanol	C_30_H_52_O	428	*P. aeruginosa*
	Octadecanal, 2-bromo	C_18_H_35_BrO	347	
22, 23	Hexadecane	C_16_H_34_	226	*B. cepacia*
	Pyrrolo[1,2-a]pyrazine-1-4-, dione, hexahydro	C_7_H_10_N_2_O_2_	154	
10	Octadecane	C_18_H_38_	254	*B. cepacia*
	Octane, 5-ethyl-2 methyl	C_11_H_24_	156	
	2-Ethyhexyl hexyl ester	C_16_H_30_O_4_	286	
	Decane, 3,8-dimethyl	C_12_H_26_	170	
17	Cyclotetracosane	C_24_H_48_	336	*B. cepacia*
	2-Ethyl-1,3,4-trimethyl-3-pyrazoline	C_8_H_14_N_20_	154	
	Cyclo(L-prolyl-L-valine)	C_10_H_16_N_2_O_2_	196	
20	1,2-Cyclopentanedione,3,5-tetramethyl	C_9_H_14_O_2_	154	*B. cepacia*
	Pyrimidinone, 4,6-amino-1,5-methyl	C_5_H_6_N_4_O_2_	154	
	Pyrimidine-2 (1H)-thione, 4,4,6-trimethyl-1-(1-phenylethyl)	C_13_H_16_N_2_S	232	
18	Cyclodecane, cyclopropane, 1-2-dibutyl	C_15_H_30_	210	*B. cepacia*
	L-leucine, N-cyclopropylcarbonyl-methyl ester	C_11_H_19_NO_3_	213	
	2,6-Dibutyl-4-methylpiperidine	C_14_H_23_N	205	
	Hexadecanoic acid, 2-hydroxyl-1-(hydroxymethyl) ethyl ester	C_19_H_38_O_4_	330	
	2,5-Cyclohexadien-1-one, 3-5-dihydr-oxy-4,4-di-methyl	C_24_H_30_O_8_	446	
21	Octadecanoic acid, 2-3-dihydroxypropyl ester	C_21_H_42_O_4_	358	*B. cepacia*
	Octadecanoic acid, 2-hydroxyl-1-(hydroxymethyl)	C_21_H_42_O_4_	358	
	l-Proline, N-allyloxycarbonyl	C_9_H_13_NO_4_	199	
	l-Alanine, N-(2-thienylcarbonyl)-pentadecyl ester	C_23_H_39_NO_3_S	409	
12	1-Acetyl-3,3-pentamethylenediaziridine	C_7_H_15_N	113	*B. cepacia*

### 3.7 Biocontrol activity of isolates and effect on plant growth

**Figure 7:**
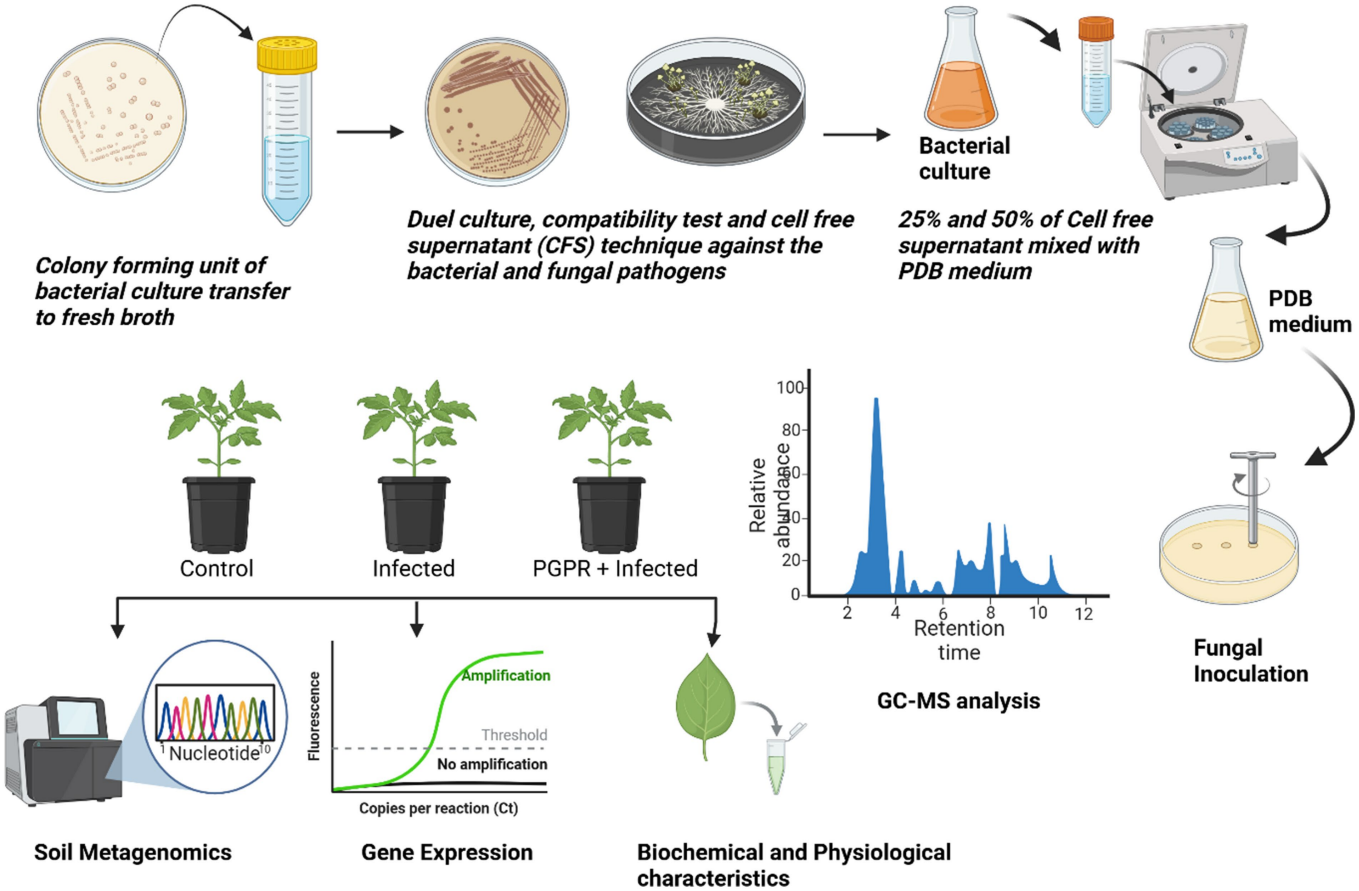
Schematic overview of the research process.

**Figure 8:**
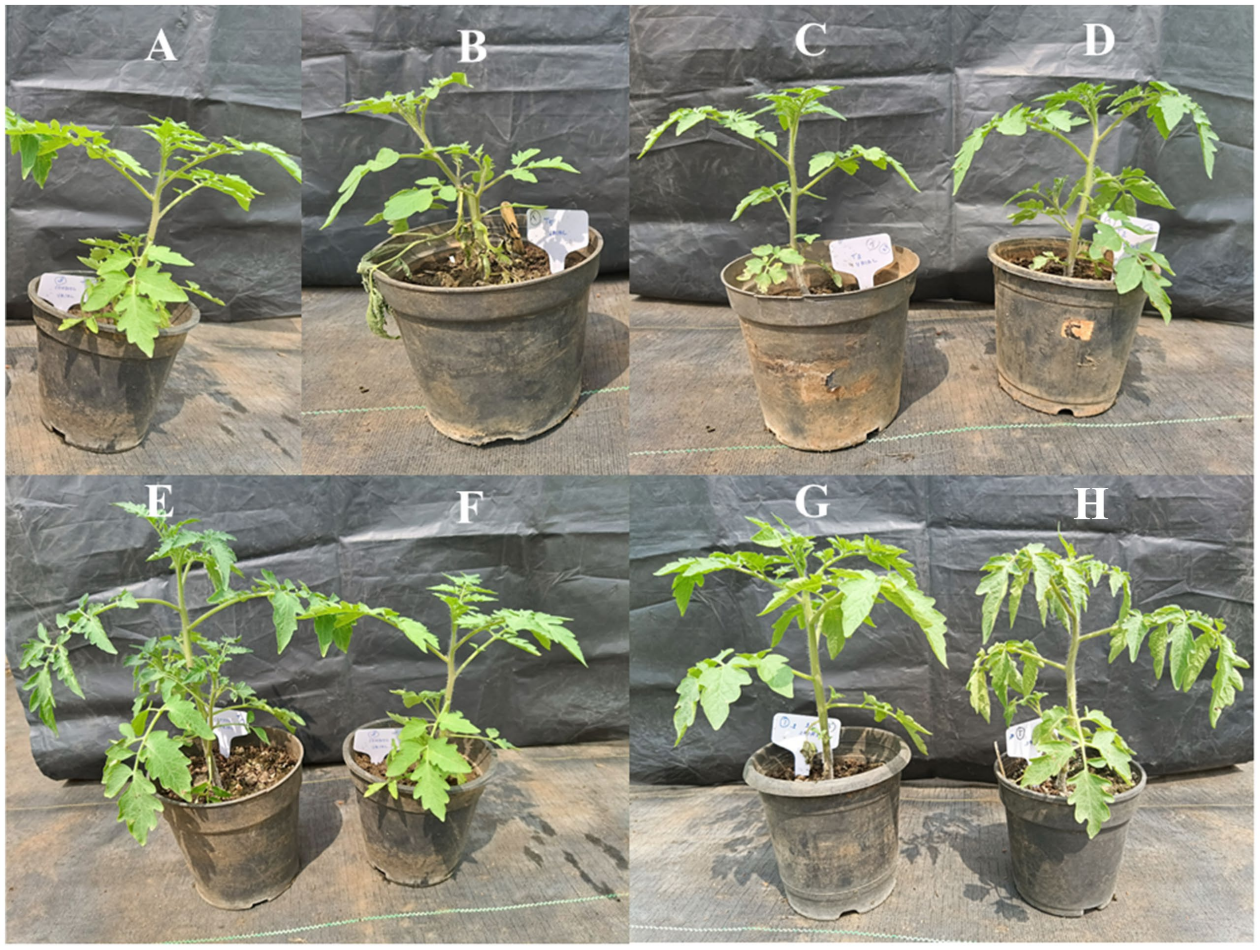
Effects of the plant growth-promoting rhizobacteria (PGPR) of *P. aeruginosa* (VITK-1) and *B. cepacia* (VITK-3) with pathogenic *F. oxysporum*. Uninoculated control **(A)**, inoculation with *F. oxysporum* **(B)**, *P. aeruginosa* **(C)**, *P. aeruginosa* was inoculated with *F. oxysporum* **(D)**, *B. cepacia* **(E)**, *B. cepacia* was inoculated with *F. oxysporum* **(F)**, inoculation of *P. aeruginosa* and *B. cepacia* **(G)**, *P. aeruginosa* and *B. cepacia* were inoculated with *F. oxysporum* **(H)**. The plants were inoculation using the root dipping method as described in section 2.6.

The individual bacterial strains and their consortia inoculation treatments improved plant growth, as illustrated in the graphical experiments in Figures 7, 8, and enhanced survival rates under diseased conditions. Soil tests were conducted before transplanting, and the detailed results are provided in Supplementary Table S3. These treatments were compared to disease control (T2) and non-inoculated control (T1). Table 6 shows that, in comparison to the individual treatments, the bacterial consortium (T7 and T8) significantly increased shoot height, along with root length, shoot diameter, fresh weight, dry weight, root fresh weight, and root dry weight. However, no significant variations in the strains in terms of physiological parameters. Treatment of T3 outperformed T5 in promoting plant growth. Inoculation with consortia (T8) notably increased plant height, root length, and survival rate (Figure 4A). Overall, bacterial treatments increased plant growth and showed clear differences compared to the diseased and non-inoculated controls. In disease severity measurements, conducted six 6 weeks after inoculation with *F. oxysporum*, shoot size, root elongation, and wilting were observed. Consortia treatments alleviated disease symptoms more effectively than individual treatments. These results confirm that consortia offer protection against *F. oxysporum in vivo*, showing a significant difference between individual strains and the consortium, as well as untreated and treated controls. These findings were further supported by gene expression analysis and metagenomic analysis, which demonstrated significant differences in microbial diversity between treated controls and treatments.

**Table 6:** Physiological parameters of plant growth with individual strains and consortia with treated (*F. oxysporum*) and untreated control.

Treatments	SL (cm)	RL (cm)	SD (mm)	FW (mg)	DW (mg)	RFW (mg)	RDW (mg)	No. of leaves
T1	35.5 ± 1.80	19.5 ± 0.5	22.8 ± 1.04	32.8 ± 0.35	10.7 ± 0.2	5.06 ± 0.5	1.8 ± 0.1	122 ± 2.6
T2	28.6 ± 3.32	11.16 ± 1.25	16.6 ± 0.55	26.1 ± 0.8	8.8 ± 0.2	3.3 ± 0.51	1.1 ± 0.1	60 ± 5
T3	56.6 ± 0.88	27 ± 2	29.9 ± 1	46.2 ± 0.5	14.9 ± 0.1	8.76 ± 0.2	3.7 ± 0.2	171 ± 3.06
T4	50 ± 1.15	22.8 ± 0.76	24.6 ± 0.55	37.9 ± 1.7	12 ± 0.15	7.6 ± 0.51	2.9 ± 0.05	145 ± 5
T5	54 ± 3.06	25.9 ± 1	27.7 ± 0.6	44.2 ± 0.5	16.9 ± 0.1	9.5 ± 0.4	5.3 ± 0.4	168 ± 2.64
T6	43.6 ± 3.21	27.9 ± 1.87	25.1 ± 0.37	38.3 ± 0.6	13.03 ± 0.15	8.5 ± 0.39	3.9 ± 0.05	152 ± 6.2
T7	64.06 ± 2.68	31 ± 1.53	32.5 ± 2.17	50.3 ± 1.5	18.1 ± 0.3	9.8 ± 0.1	6.1 ± 0.2	195 ± 5
T8	60.1 ± 2.15	29.6 ± 0.78	30.2 ± 0.4	48.8 ± 0.7	19.2 ± 0.5	9.63 ± 0.55	5.7 ± 0.25	201.6 ± 7.6

The data table represents the different treatments of mean and standard deviation of T2, T4, T6, and T8 vs. control. The different letters show a statistically significant difference between the treatments according to the Student’s *t*-test, and a comparison test was performed on Duncan’s multiple tests, respectively. SL, shoot height (cm); RL, root length (cm); SD, shoot diameter; FW, fresh weight; DW, dry weight; RFW, root fresh weight; RDW, root dry weight.

### 3.8 Analysis of plant growth chlorophyll and protein content

**Figure 9:**
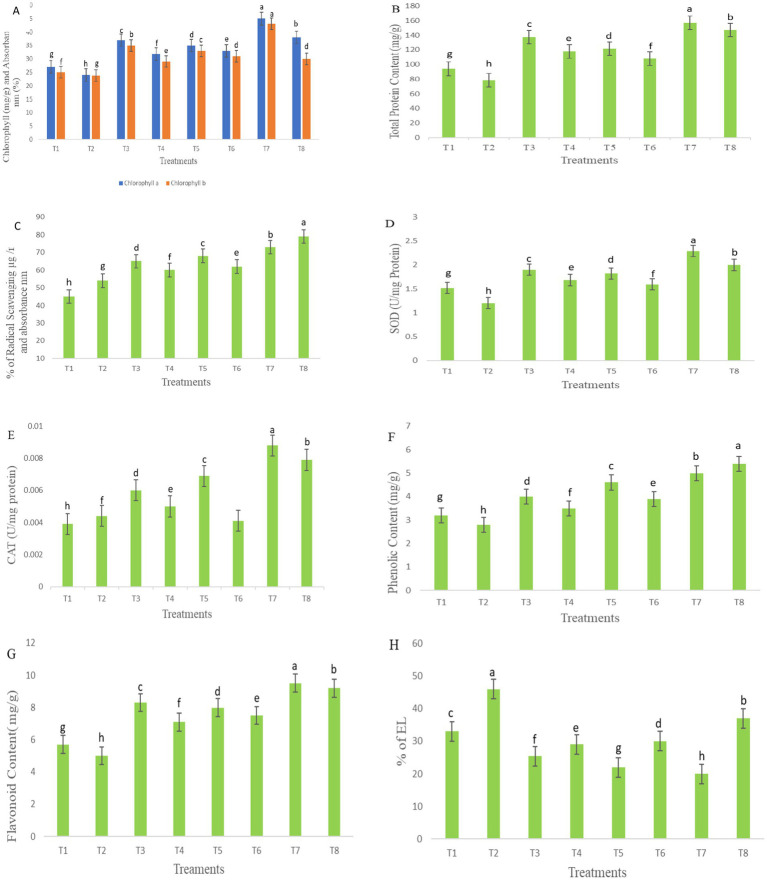
Chlorophyll **(A)**, total protein content **(B)**, antioxidant activity **(C)**, SOD **(D)**, CAT **(E)**, phenolic content **(F)**, flavonoid content **(G)**, and electrolyte leakage **(H)**. The table represents the bacterial treatments as individual and consortia against the treated (*F. oxysporum*) and untreated control. The letters indicate the significant differences between the treatments and control groups. The mean ± standard deviation shows, according to the Student’s *t*-test and comparison, Duncan’s multiple tests. The error bar represents the mean and standard error of the one-way ANOVA test; *p* < 0.005, *p* < 0.001, and *p* < 0.0001.

Total chlorophyll content increased in all treatments, including bacterial treatments applied to disease-affected plants, as illustrated in Figure 9A. Notable and significant differences in chlorophyll content were observed in bacterial-treated plants compared with the diseased control group. Significant variation was observed in both total chlorophyll a and b content. Among the treatments, T7 exhibited a higher chlorophyll content, increasing by 66.6 mg/g, followed by T8, which showed an increase of 58.3 mg/g, as compared to other groups. Similarly, chlorophyll a concentration was significantly higher in infected seedlings treated with T4, which showed a 32.5 mg/g increase, and T6, with a 37.5 mg/g increase. Chlorophyll b content also showed a similar enhancement in these treatments, with T4 showing a 21.8 mg/g increase and T6 at 30.2 mg/g also exhibiting higher values. The total protein content in *S. lycoperisum* showed a high concentration of soluble protein under biotic stress, as determined by Lowry’s method. A significant increase in protein concentration was observed in T8, which reached 87.6 mg/g after the inoculation of *F. oxysporum* (Figure 9B). Similarly, treated samples showed high protein content peaks, with T4 increasing by 50 mg/g and T6 by 37.5 mg/g compared to the diseased control. These findings show that seedlings treated with bacterial consortia had significantly higher protein content than both treated and untreated controls, respectively.

### 3.9 Antioxidant activity of SOD and CAT

The antioxidant activity was assessed based on the absorbance of color formation, which is directly proportional to the concentration of free radicals. Under stress conditions, lower concentrations of antioxidants were observed in T7, with a reduction of 73%, followed by T4 at 60%, and T6 at 62%, corresponding to higher absorbance values, as illustrated in Figure 9C. Among the treatments, T8 demonstrated higher enzymatic activity, increasing by 79% in leaf extracts inoculated with *F. oxysporum*, however, no significant differences were found among the inoculation treatments in the seedlings under biotic stress.

Bacterial inoculation reduced the activity of SOD in *F. oxysporum*-inoculation leaf samples, with the greatest reduction observed in T4 at 40 mg/g, followed by T5 at 31.2 mg/g, T6 at 32.5 mg/g, and T8 at 66.6 mg/g, as shown in Figure 9D. Conversely, stress enzyme activity was highest in T7 (50.6 mg/g) compared to other treatments under non-stress conditions. These SOD enzyme activity levels closely resembled those of CAT activity, measured in U/mg protein. Notably, T5 exhibited a CAT activity increase of 55 mg/g, while T7 showed the highest CAT activity at 70 mg/g, both significantly higher than SOD activity. However, no significant differences were observed between bacterial treatments in the activity of stress enzymes in fresh leaf samples under stress conditions, as presented in Figure 9E.

### 3.10 Phenolic and flavonoid compounds

Phenolic and flavonoid contents were observed under bacterial inoculation and compared with *F. oxysporum*-induced stress conditions, as illustrated in Figures 9F,G. In seedlings, inoculation with *F. oxysporum*, treatments with *P. aeruginosa* and *B. cepacia* in the consortium (T8) exhibited the highest phenolic content about 82.7 mg/g fresh weight and flavonoid content at 84 mg/g, followed by T4, which ranged from 20.6 mg/g to 42.2 mg/g, and T6, which ranged from 34.4 mg/g–50 mg/g. Other treatments also showed significant variations, with phenolic content measured at 25.1 mg/g in T3, 43.7 mg/g in T5, and 56.2 mg/g in T7. Flavonoid concentrations were higher in treatments under non-*F. oxysporum* stress, with T3 at 45.61 mg/g, T5 at 40.2 mg/g, and T7 at 66.6 mg/g demonstrating the most pronounced increases. These results suggest that bacterial inoculation treatments significantly influenced phenolic and flavonoid production, with marked differences observed among the groups under stress.

### 3.11 Electrolyte leakage

Electrolyte leakage serves as an indicator of cell membrane stability under stress conditions. As illustrated in Figure 9H, seedlings inoculated with *F. oxysporum* exhibited relatively high electrolyte leakage, with T2 at 46%, T4 at 29%, T6 at 30%, and T8 at 37%. However, no significant differences were observed between bacterial treatments and the treated control. In contrast, treatments T3, T5, and T7 significantly reduced electrolyte leakage following *F. oxysporum* inoculation, with values recorded at 25, 22, and 20%, respectively, compared to the control groups.

### 3.12 Defense-regulating gene expression analysis against *Fusarium oxysporum* infection

**Figure 10:**
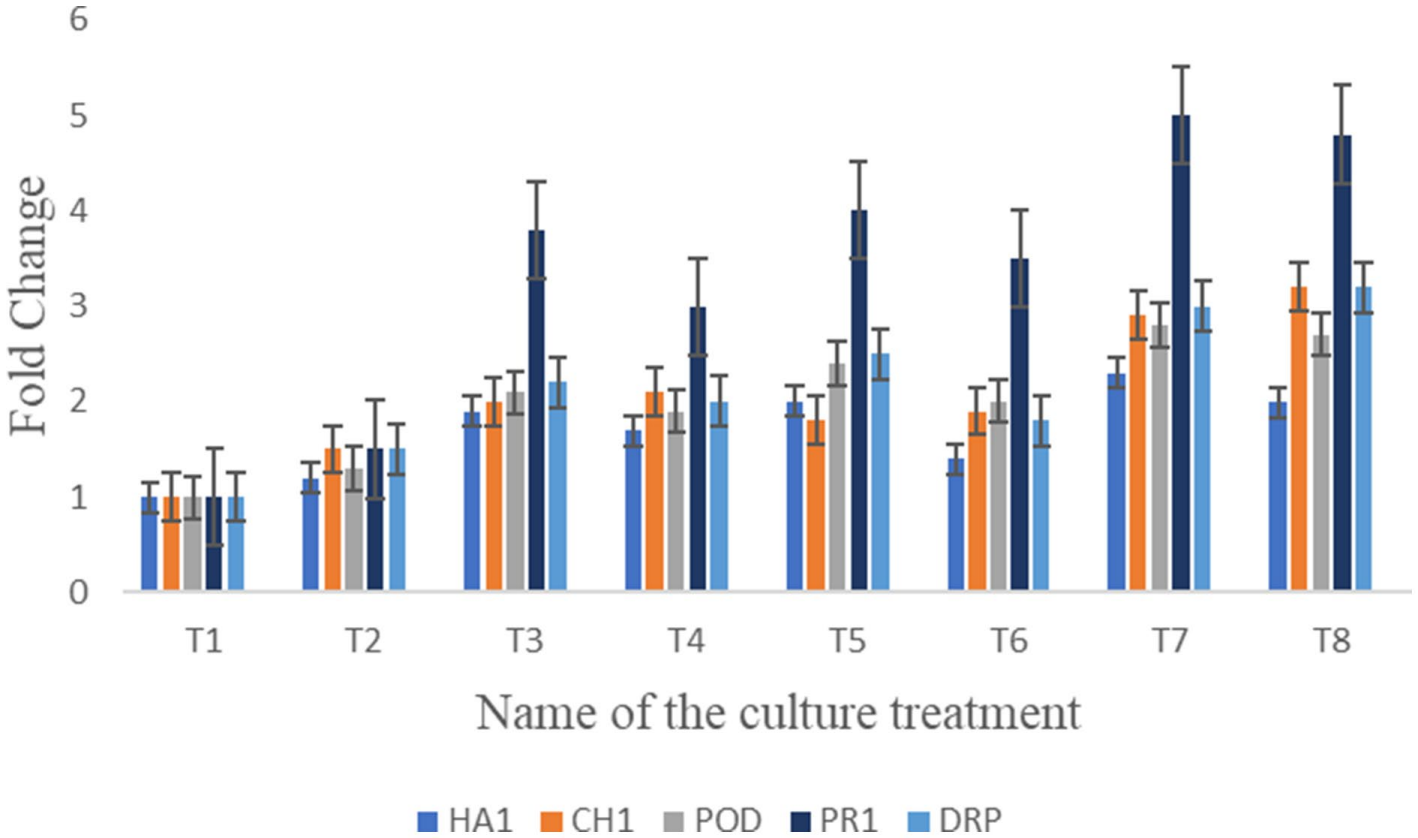
Illustration of bar graph shows the relative gene expression analyzed of *HA1*, *CH1*, *POD*, *PR1*, and *DRP* in seedlings under bacterial treatments against the plant pathogens.

The expression of defense-regulating genes (*HA1*, *CHI*, *POD*, *PR1*, and *disease-resistant protein*) was analyzed in treated plant roots under *F. oxysporum* infection using a Student’s *t*-test (Figure 10). This experiment assessed the ability of bacterial treatments to protect seedlings from fungal infection. All genes were upregulated in treatments T7: T8: T5: T4, with fold changes ranging from *PR1* (5), disease-resistant protein (3.2), *POD* (2.4), and *CHI* (2), respectively. Among the genes, *POD* and *PR1* were highly upregulated in plants treated with bacteria post-fungal infection. *HA1* and *CHI* showed moderate fold changes between 1.7 and 1.9. Notably, the consortium treatment significantly upregulates the expression of *PR1*, *CHI*, and Disease-resistant protein (*DRP*) compared to the treated control. However, no discernible difference was observed between the treatments in root samples following fungal infection. Supplementary Data S4 includes individual Cq values, amplification efficiencies, and melt-curve diagnostics.

### 3.13 Metagenomic diversity of soil microbiome under treatments

**Figure 11:**
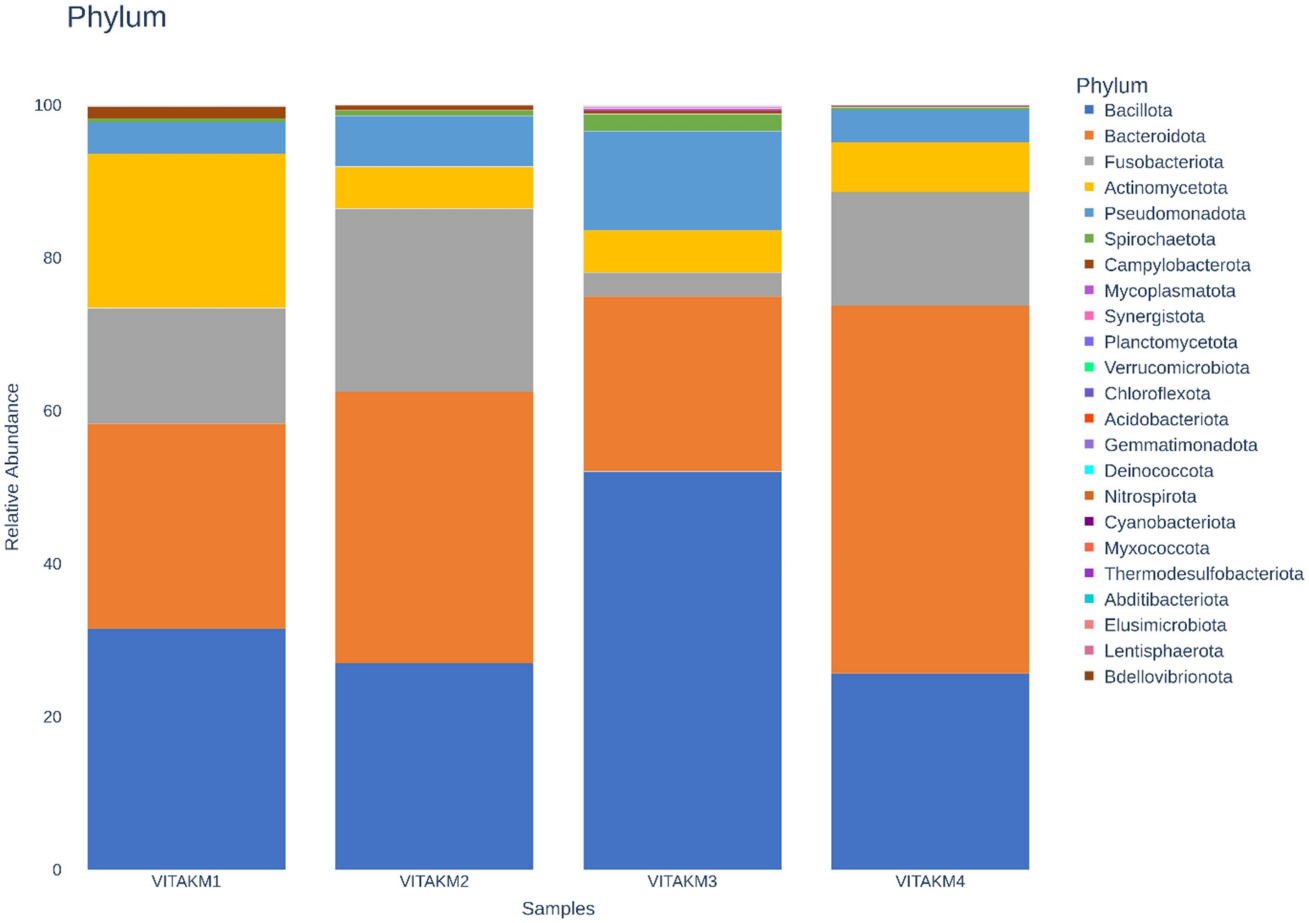
Phylum-based taxonomy communities of untreated and treated control followed by consortia with fungal infection were analyzed using 16S amplicon datasets. The picture clearly shows the presence of bacteroidota in the soil community among the treatments. The consortia with fungal stress exhibit more than 70% of the microbiome which promotes soil quality compared to other samples.

Figure 11 illustrates the metagenomic sequencing analysis of soil samples, which were coded as VITAKM1, VITAKM2, VITAKM3, and VITAKM4. A comparative analysis of soil metagenomic data revealed significant shifts in microbiome diversity under bacterial inoculation and treated control conditions. The untreated control samples predominantly contained phyla such as *Actinomycetota*, *Campylobacterota*, *Pseudomonadota*, and *Bacteroidota*. In contrast, treated samples displayed a higher abundance of *Bacteroidota*, *Bacillota*, *Fusobacteriota*, *Pseudomonadota*, *Cyanobacteriota*, *Spirochaetota*, *Acidobacteriota*, and *Mycoplasmatota*. Although some phyla were common across both groups, unique microbiome profiles with higher abundances were observed in the treated samples, particularly in the PKM1 variety. These findings suggest that bacterial inoculation enriched the soil microbiome and stimulated soil quality by promoting beneficial microbes like *Bacteroidota*, which support plant growth and stress resistance.

**Figure 12:**
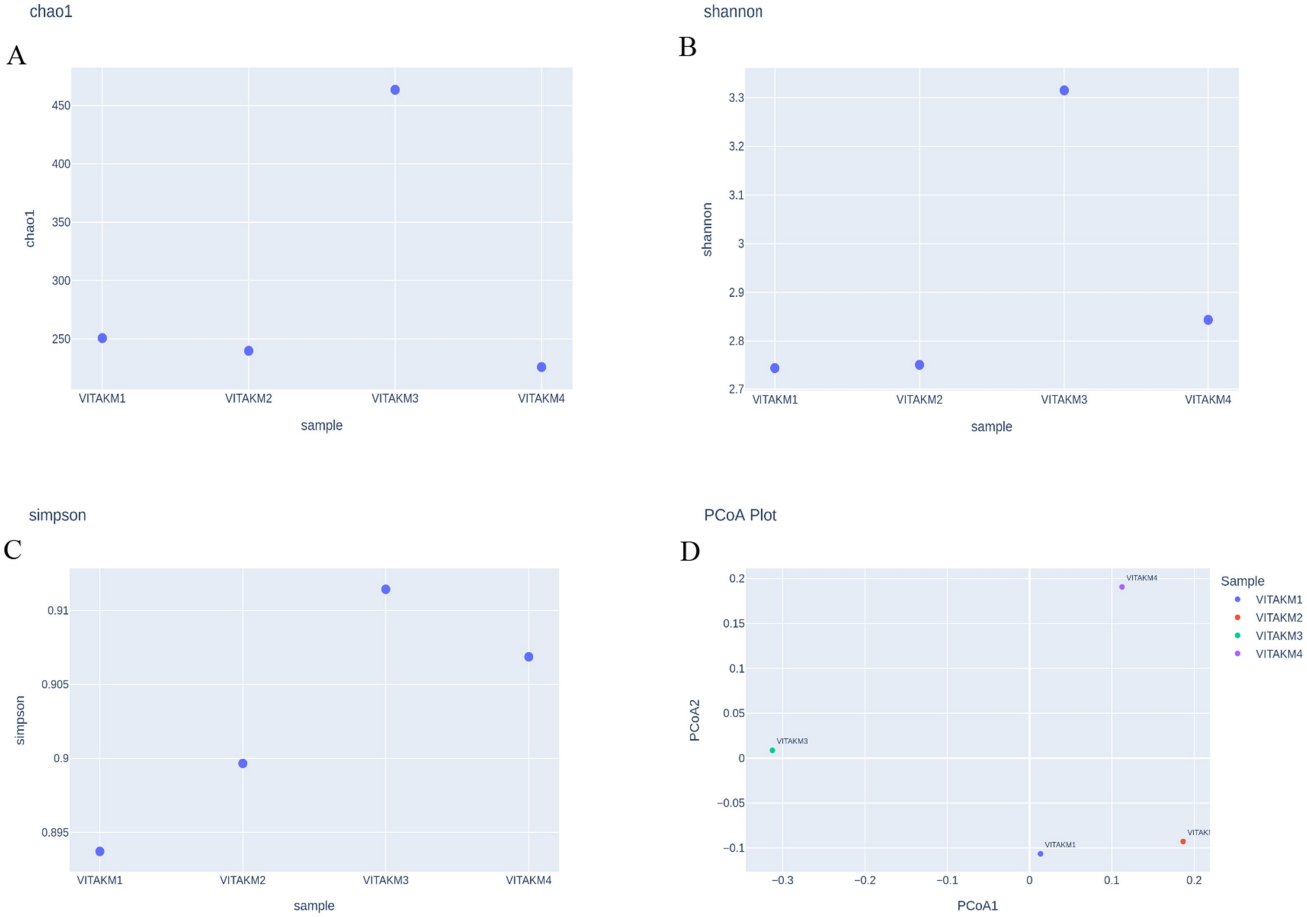
Alpha diversity of microbiome species richness **(A)** chao1; **(B)** shannon; **(C)** simpson; **(D)** PCoA Plot.

Alpha diversity, which measures species richness and evenness, was analyzed using bioinformatics tools. The richness statistics, including Chao1, Shannon, and Simpson indices, are illustrated in Figures 12A–C, indicating higher microbial diversity in the treated samples than in the controls. Boxplots further depict the distribution of microbial species diversity in the treated soil. Principal coordinate analysis (PCoA) was used to perform the beta diversity analysis, which is illustrated in Figure 12D, highlighting distinct differences in microbial abundance and diversity between treatments and control samples.

**Figure 13:**
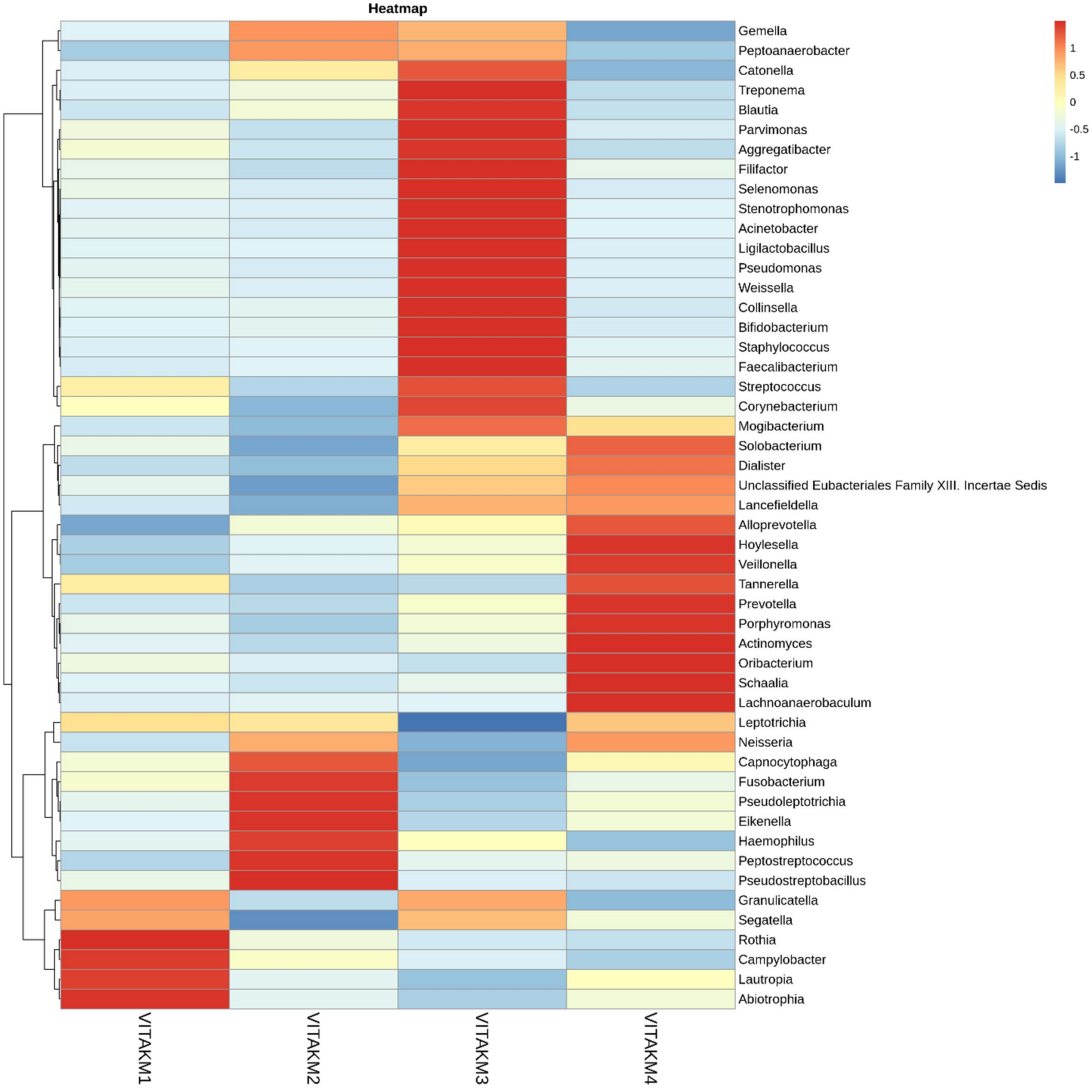
List of bacterial metabolisms in the soil of tomato plants with treated and untreated groups on heatmap analysis.

**Figure 14:**
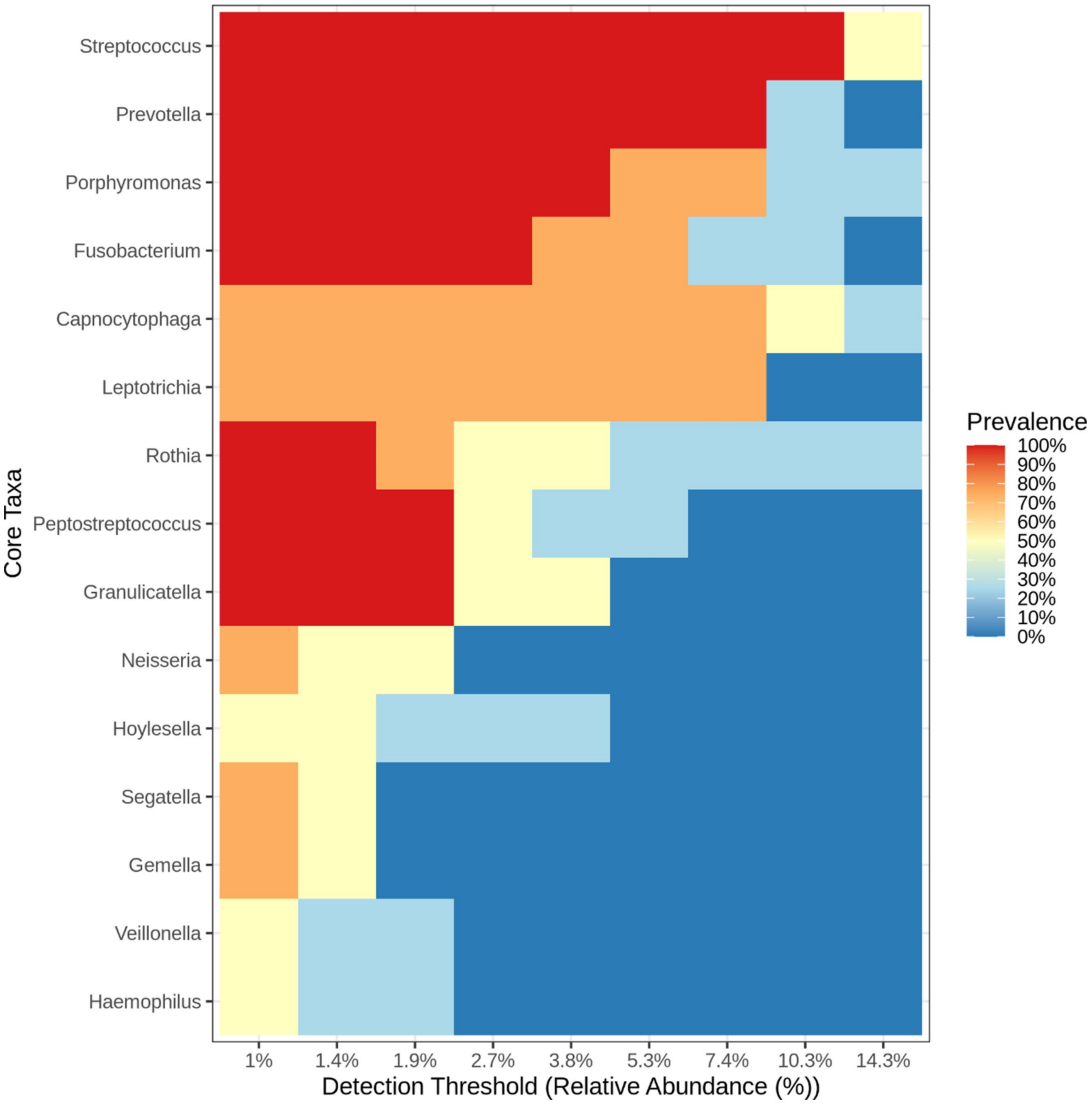
The heat map listed the core microbiome bacteria on the soil of tomato plants (treated and untreated groups).

Heatmap and core microbiome diversity: The Heatmap presented in Figure 13 illustrates the samples’ relative abundance of microbiome genera, revealing significant differences between treated and control groups. Treated samples, particularly VITAKM3 and VITAKM4, showed an increased abundance of bacterial genera. The core microbiome analysis, shown in Figure 14, revealed that *Streptococcus*, *Fusobacterium*, and *Porphyromonas* were the most prevalent genera, while *Veillonella*, *Neisseria*, and *Haemophilus* had the lowest prevalence. These findings indicate that bacterial inoculation significantly altered the soil microbiome, enhancing microbial diversity and promoting beneficial microbial populations.

## 4 Discussion

This study established and characterized two plant growth-promoting rhizobacteria (PGPR) strains, *P. aeruginosa* and *B. cepacia*, along with their combination (consortium), for their biocontrol activity against phytopathogens under both *in vitro* and *in vivo* conditions. These strains produce bioactive compounds through their culture filtrates, effectively suppressing pathogenic microbes (Heo et al., 2022; Kalyandurg et al., 2022). Phylogenetic analysis confirmed the taxonomic identity, highlighting their potential to compete with phyopathogens in the rhizosphere.

Recent studies have demonstrated that individual PGPR strains can suppress phytopathogens under various experimental and environmental conditions, particularly in response to biotic stress (Kashyap and Manzar, 2025; Kong et al., 2020; Mehmood et al., 2023; Xie et al., 2023). However, limited research has explored the synergistic role of bacterial consortia under stress conditions. In studies assessing microbial diversity, plant growth, and nutrient uptake, PGPR consortia outperformed individual strains, effectively managing pathogenic stress and enhancing rhizosphere health (Nafady et al., 2019; Vishwakarma et al., 2020; Yaduwanshi et al., 2021). Recent reviews have further demonstrated that a consortium of three PGPR strains can significantly enhance the synthesis of secondary metabolites and modulate the expression of stress-responsive genes (Saikia et al., 2018), emphasizing the relevance of the soil microbiome in achieving sustainable and high-quality crop production (Chaudhary et al., 2023). Ferrante et al. (2023) also reported that such microbial interactions contribute to improved plant metabolic functions and growth pathways, as evidenced by quantitative synthesis in meta-analyzes.

Building on these findings, the present study investigated the physiological and biochemical responses of tomato seedlings exposed to bacterial consortia, particularly under biotic stress conditions. Moreover, this research provides insights into metagenomic profiling of the soil microbial dynamics (pH 7.8) and the expression of defense-related genes in treated tomato plants.

These bacterial strains, *Pseudomonas aeruginosa* and *Burkholderia cepacia*, are known for effective root colonization, promoting plant development, and suppressing soil-borne fungal and bacterial pathogens through plant growth-promoting (PGPR) traits such as phosphate solubilization, indole-3-acetic acid (IAA) production, siderophore chelation, and hydrogen cyanide (HCN) synthesis (Choudaker et al., 2024; Kashyap et al., 2021; Singh et al., 2022). Nitrogen fixation screening revealed that VITK-1 and VITK-3 are capable of fixing atmospheric nitrogen in the rhizosphere, and additionally, they effectively manage root-knot diseases, functioning as potential biocontrol agents that enhance crop productivity (Liu et al., 2022; Sanow et al., 2023). These strains are also capable of inducing systemic resistance (ISR), demonstrating unique potential compared to other beneficial strains of similar genera by regulating phytohormone signaling, producing secondary metabolites, and synthesizing osmoprotectants (Anjali et al., 2023; Jalmi and Sinha, 2022).

In antagonistic activity, both VITK-1 and VITK-3 exhibited strong inhibitory activity against test pathogens in both *in vivo* and *in vitro* conditions. VITK-1 showed the highest suppression of *F. oxysporum*, *R. solanacearum*, *S. protearum* followed by *C. canascens*, and *V. dahlia*. This suppression was mediated through the production of bioactive compounds and the activity of hydrolytic enzymes (Xie et al., 2023). Similar findings have been reported by Siddiqui et al. and Satya et al., where *P. aeruginosa* and *Burkholderia* spp. significantly reduced root rot and leaf spot diseases by inhibiting mycelial growth and spore formation. However, *in vivo* studies often encounter limitations such as environmental variability and inconsistent pathogen pressure (Pandey et al., 2018). In the present study, the bacterial consortium significantly suppressed *F. oxysporum* under *in vivo* conditions, reducing disease symptoms and improving seedling survival rates, as indicated by the disease index.

Greenhouse inoculation with the VITK-1 and VITK-3 consortium significantly increased plant growth parameters. Similarly, earlier studies have demonstrated that *P. aeruginosa RTE4* strains promote plant growth traits and elevate enzymatic defenses against phytopathogens (Chopra et al., 2020). In the present study, the bacterial strains formed biofilms in the rhizosphere in response to test pathogens, thereby protecting the root environment and promoting root elongation, activating the plant’s immune system. These findings are consistent with previous reports by Ambreetha and Balachandar (2022) and Paungfoo-Lonhienne et al. (2016), thereby validating the present observations. Moreover, biosurfactant production and drought tolerance assays confirmed the contribution of *P. aeruginosa* and *B. cepacia* to plant stress resilience and growth enhancement. Significant increases were observed in root length, as well as in fresh and dry shoot biomass, following bacterial consortium treatment.

Under biotic stress imposed by *F. oxysporum*, biochemical parameters, such as total protein content and antioxidant activity, were significantly elevated following consortium inoculation. These PGPR strains indirectly inhibit pathogenic organisms by producing cell wall-degrading enzymes (Gul et al., 2023). Such biochemical changes directly supported seedling growth and were associated with enhanced oxidative stress responses, including reduced electrolyte leakage. Previous studies have reported that *Bacillus amyloliquefaciens* NJN-6 enhances structural integrity, inhibits spore germination, and stimulates protein synthesis by producing antifungal compounds (Yuan et al., 2012). The accumulation of superoxide dismutase (SOD) and catalase activities was significantly higher in consortium-treated plants inoculated with *F. oxysporum* compared to both the inoculated control and individual bacterial treatments. These findings are further supported by Alharbi et al. (2022), who reported that PGPR strains *Pseudomonas koreensis* MG209738 and *Bacillus coagulans* NCAIM B.01123 reduced oxidative stress indicators and facilitated reactive oxygen species (ROS) detoxification.

Furthermore, PGPR-treated plants exhibited significantly higher chlorophyll content, counteracting the typical decline observed under biotic stress conditions. These results confirm that PGPR strains enhance growth parameters and photosynthetic pigment content compared to uninoculated controls (Gashash et al., 2022). In addition, the *P. aeruginosa* and *B. cepacia* consortium significantly increased phenolic and flavonoid content in tomato leaf extracts, thereby enhancing plant resilience and stimulating secondary metabolite production against pathogens (Palermo et al., 2025). Biochemical characterization further supported the biocontrol potential of consortium inoculation. Culture filtrate analysis indicated that ethyl acetate and hexane were the most effective solvents for extracting antifungal bioactive compounds, consistent with findings by Heo et al. (2022). Identified bioactive compounds included hexadecane, octadecane, cyclopropane, pyrimidine, and ethyl iso-allocholate—each of which has been previously associated with antifungal activity and plant growth promotion (Alotaibi et al., 2022; El-Gendi et al., 2022).

Despite the successful *in vivo* biocontrol activity, the combined bacterial strains provided significant protection against *F. oxysporum*. Gene expression analysis further supported this effect, which revealed the upregulation of five key defense-related genes. Specifically, *HA1*, *CH1*, *POD*, *PR1*, and *DRP* expression levels were markedly elevated in consortium-treated plants compared to untreated controls. Among these, *PR1* was notably upregulated and is known to be closely associated with induced systemic acquired resistance, as observed in *P. fluorescens WCS417r* inoculation (Beneduzi et al., 2012). The *CHI* (chitinase) gene showed upregulated in root tissues, highlighting its critical role in plant-pathogen interactions under biotic stress (Xuan et al., 2024). Furthermore, *HA1*, *POD*, and *DRP* genes, which are linked to nutrient uptake and pathogenesis-related proteins activity, were also upregulated, indicating enhanced physiological and metabolic responses in tomato plants (El-Gendi et al., 2022; El-Maraghy et al., 2020; Kiddee et al., 2024).

Metagenomic profiling of PGPR-treated rhizosphere soils revealed microbial gene communities involved in the biosynthesis of secondary metabolites, which suppress phytopathogens and modulate plant stress responses (Adedayo et al., 2023). According to Raza et al. (2016), microbial communities suppress *Ralstonia solanacearum*, the causative agent of bacterial wilt, activating the plant immune system and inducing the expression of defense-related genes. Our study confirmed these findings that PGPR inoculation enhanced plant growth by upregulating genes associated with nutrient uptake and defense mechanisms, particularly in root tissues.

Hence, the study revealed that metagenomics analysis showed significant shifts in microbial diversity under consortium inoculation with *F. oxysporum* and the corresponding treated controls. In consortium-treated samples, the dominant phyla were *Bacteroidota*, *Bacillota*, *Pseudomonadota*, and *Spirochaetota*. In contrast, the control sample mainly contained *Bacteroidota* and *Pseudomonadota*, which are commonly represented in the natural soil microbiome. Notably, *Bacteroidota* and *Bacillota* were significantly more dominant in consortium-treated soils than in control samples, indicating the consortium’s effective modulation of rhizosphere microbiomes under biotic stress. Similar rhizosphere microbial compositions have been previously reported in responses to PGPR inoculation and their competitive interactions under stress (Bigatton et al., 2024). In efforts to combat *Meloidogyne graminicola*, Samain et al. (2023) reported that PGPR significantly altered rhizosphere microbial communities, contributing to a 37.8 to 47.9% improvement in wheat growth. While the extract microbial community composition may vary depending on bioinoculants and experimental conditions, our findings suggest that PGPR consortia have the potential to reshape the availability and activity of microbial amendments in the soil rhizosphere. According to Kruczyńska et al. (2023) and Zhang et al. (2019), the high relative abundance of these microbes in alkaline pH conditions (ranging from 6.74 to 7.94) is critical for improving soil quality. The PGPR consortia enhanced the soil’s chemical and biological properties, as supported by earlier studies focusing on microbial contributions to soil health. Moreover, previous research (Singh et al., 2023) has emphasized that microbial diversity positively correlated with improved soil fertility. Interestingly, *Fusobacteriota* was more prevalent in consortium-treated soils with *F. oxysporum* stress, suggesting that bacterial consortia enhanced microbiome bioavailability without disrupting the natural soil balance.

Therefore, our study demonstrates the functional role of bacterial consortia in promoting plant growth and development under biotic stress conditions, highlighting their potential as effective biocontrol agents. Through the production of biocontrol compounds and suppression of fungal and bacterial pathogens via culture filtrate, the bacterial isolates contribute to enhanced plant protection. Inoculated treatments significantly reduced disease symptoms, reinforcing the PGPR consortia’s efficacy in mitigating pathogen-induced stress and supporting healthy plant growth.

## 5 Conclusion

This study revealed that applying a two-strain bacterial consortium substantially increased seedling growth and survival by improving physiological and biochemical traits under *F. oxysporum* stress. The results emphasize the crucial role of microbial diversity, notably from the phyla *Bacteroidota* and *Pseudomonadota*, in increased nutrient bioavailability for tomato seedlings. Additionally, the genomic analysis of treated plants further elucidated plant-microbiome dynamics in response to pathogenic infection. It supports the use of microbial inoculants as a safe and environmentally friendly strategy in sustainable agriculture. However, further research is needed to optimize various microbial consortia formulations for improving yield, resilience, and overall productivity in tomato cultivation across different soil conditions.

## Data Availability

The original contributions presented in the study are publicly available. This data can be found at: https://www.ncbi.nlm.nih.gov, accession numbers SAMN47269860, SAMN47269861, SAMN47269862, SAMN47269863, and PRJNA1233473.

## Glossary

PGPR: Plant growth-promoting rhizobacteria
ISR: Induce systemic resistance
GC-MS: Gas chromatography-mass spectrometry
NaCl: Sodium chloride
CPG: Casamino acid-peptone-glucose
CFS: Cell free supernatant
PDA: Potato dextrose agar
CFU: Colony forming unit
PEG: Polyethylene glycol
MSM: Minimal salt medium
NH_4_Cl: Ammonium chloride
KH_2_PO_4_: Potassium dihydrogen phosphate
Na_2_HPO_4_: Disodium hydrogen phosphate
MgSO_4_: Magnesium sulfate
dH_2_O: Distilled water
HPLC: High-performance liquid chromatography
NAF: National Agro Foundation
BSA: Bovine serum albumin
DPPH: 2,2-Diphenyl-1-picrylhydrazyl
SOD: Superoxide dismutase
EDTA: Ethylenediamine tetraacetic acid
PVP: Polyvinylpyrrolidone
Na_2_CO_3_: Sodium carbonate
K_2_HPO_4_: Potassium phosphate dibasic
AlCl_3_: Aluminum chloride
EC: Electrical conductivity
qRT-PCR: Quantitative reverse transcription polymerase chain reaction
RNA: Ribonucleic acid
DNA: Deoxyribonucleic acid
CDNA: Complementary DNA
BLAST: Basic Local Alignment Search Tool
NCBI: National Center for Biotechnology Information
SRA: Sequence Read Archive
PCoA: Principal coordinate analysis
ANOVA: Analysis of variance
