# Supplementary material for: Bio-priming of tomato seedlings with bacterial consortium against Fusarium oxysporum: a study on morphological parameters and molecular profiling
Source: Front Microbiol. 2025 Jul 9;16:1606896. doi: 10.3389/fmicb.2025.1606896 (PMC12283285; doi:10.3389/fmicb.2025.1606896)
Supplement: Supplementary file 1 [file Data_Sheet_1.pdf]

Bio-Priming of Tomato Seedlings with Bacterial Consortium against *Fusarium oxysporum*: a study on Morphological Parameters and Molecular Profiling

Supplementary Material

Supplementary Data S1. Antioxidant activity of DPPH Equation

$$\text{DPPH percentage (\%) inhibition} = [(A_B - A_A)/ A_B] \times 100$$

A_B = Denotes the absorbance of DPPH radical + methanol (control)

A_A= Represents the absorbance of DPPH radical + sample extract

Supplementary Table S2. List of Primer Sequences of Gene Expression

Gene	Primer Sequence (5'-3')	Product Size	Gene function
HA1	GAACCCTTCATGGGCTCCAA<br>GCAACTCACGTAGCCTAGCA	126bp	Role in gene regulation, cell division, and plant growth and development
CHI	TAGCTTCGGTGCTTCCATCT<br>GCACATGGGAACTCTGGTTT	159bp	Regulation of plant defense mechanism under stress conditions
POD	ACCAACAGACCAGACCCAAG<br>CGAACGTGTTGCTGCTGTAT	244bp	Role of the peroxidase enzyme in plant defense, wound healing, and stress response
pathogenesis-related protein-1	TGGGACAAGAGACTAGCACG<br>TGAGAGGGGTTCCACCTAGT	134bp	Inducing the systemic acquired resistance and boosting the plant immune system
Disease-resistant protein RPV1	TCAAGGGCAAGAGGTTCCAA<br>GGACCAACTTCCTTGCCTGA	141bp	Triggering immune responses
Actin	TAAAAGTGCGAGTGTCCTGT<br>CAGGCACCTCTCAAGTATGT	150bp	Housekeeping gene

Supplementary Table S3. Characterization of Soil Sample

S. No.	Parameter	Unit	Results
1	pH	-	7.90
2	Electrical Conductivity	mS/cm	0.20
3	Organic Matter	%	1.18
4	Nitrate Nitrogen	mg/kg	53.38
5	Available Phosphorus	mg/kg	9.89
6	Potassium Exchangeable K	mg/kg	16

7	Calcium Exchangeable Ca	mg/kg	2295
8	Magnesium Exchangeable Mg	mg/kg	1159
9	Sodium Exchangeable Na	mg/kg	386
10	Sulfur Available S	mg/kg	25.98
11	Zinc Available Zn	mg/kg	0.37
12	Manganese Available Mn	mg/kg	5.18
13	Iron Available Fe	mg/kg	12.70
14	Copper Available Cu	mg/kg	2.45
15	Boron Available B	mg/kg	0.4
16	Cation Exchange Capacity (by addition)	meq/100g	22.85
17	K Saturation	%	0.18
18	Ca Saturation	%	50.21
19	Mg Saturation	%	42.26
20	Na Saturation	%	7.34

Supplementary Table S3. Expression of Defense Genes through qRT-PCR (Amplification, Melting, Cq values)

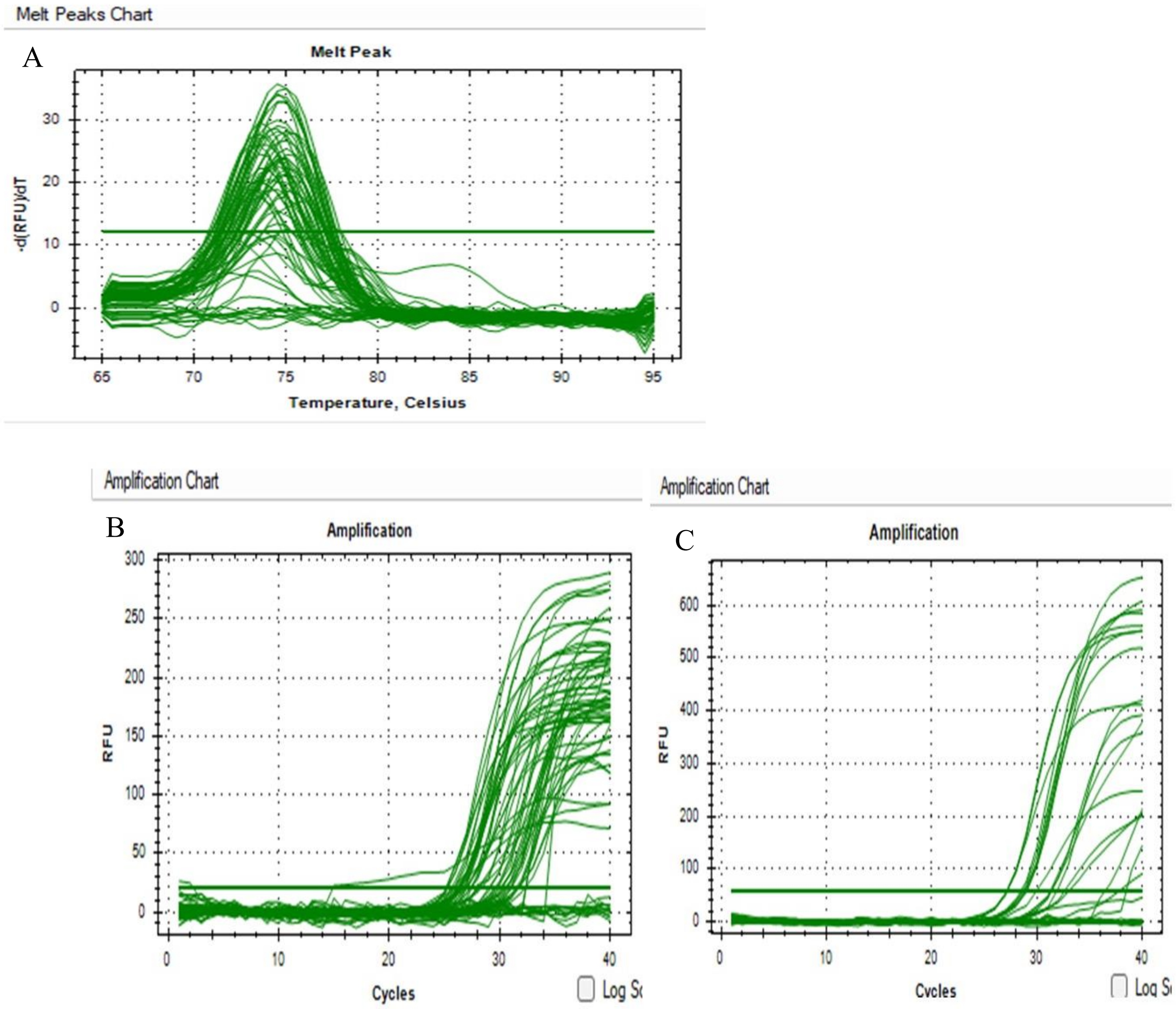

D

Treatment	HA1	Cq value	CH1	Cq value	POD	Cq value	PR1	Cq value	DRP	Cq value
T1	1	25.00	1	25	1	25	1	25	1	25
T2	1.50	24.737	1.30	24.415	1.30	24.621	1.5	24.415	1.50	24.415
T3	2.00	24.075	2.10	24	2.10	23.930	3.8	23.075	2.20	23.863
T4	2.10	24.235	1.90	23.930	1.90	24.075	3	23.415	2	24
T5	1.80	24.00	2.40	24.153	2.40	23.737	4	23	2.50	23.678
T6	1.90	24.515	2	24.075	2	24	3.5	23.193	1.80	24.153
T7	2.90	23.799	2.80	23.464	2.80	23.515	5	22.678	3	23.415
T8	3.20	24.007	2.70	23.322	2.70	23.568	4.8	22.737	3.20	23.322
